# Hyaluronic Acid-Functionalized Bismuth Vanadate/Molybdenum Disulfide Nanoheterojunctions Achieve Efficient Phototherapy of Hypoxic Tumor

**DOI:** 10.34133/bmr.0228

**Published:** 2025-07-18

**Authors:** Yunqing Pang, Jia Guo, Qianlong Ma, Jing Qi, Lv Liu, Yinzhong Bu, Jing Wang

**Affiliations:** ^1^ The First School of Clinical Medicine, Lanzhou University, Lanzhou, Gansu 730000, China.; ^2^ School/Hospital of Stomatology, Lanzhou University, Lanzhou, Gansu 730000, China.; ^3^ Clinical Research Center for Oral Diseases, Lanzhou, Gansu 730000, China.

## Abstract

Photodynamic therapy (PDT) is a promising cancer treatment modality due to its minimally invasive nature and spatiotemporal selectivity. However, its effectiveness is substantially hindered by tumor hypoxia. In this study, bismuth vanadate/molybdenum disulfide@hyaluronic acid (BiVO_4_/MoS_2_@HA, BM@HA) nanoparticles were engineered to overcome the challenges of tumor hypoxia in PDT. The formation of p–n heterojunctions between MoS_2_ and BiVO_4_ facilitated electron transfer from MoS_2_ to BiVO_4_, imparting BM@HA with photothermal properties in the near-infrared (NIR) region and achieving an improved photothermal efficiency of 51.9%. After 808-nm laser irradiation, the electron transfers and the energy generated by photothermal effects enhanced the separation of electron–hole pairs in BM@HA, leading to the production of reactive oxygen species and the hydrolysis of oxygen. Animal experiments revealed the strong tumor-targeting capability of BM@HA, as shown by tumor photothermal imaging and in vivo small-animal imaging. Following 808-nm laser irradiation, it enabled precise tumor phototherapy by combining PDT with photothermal therapy. Furthermore, proteomic analysis revealed that BM@HA + NIR may induce necroptosis of tumor cells by activating peptidylprolyl isomerase D-related pathways. In summary, the BM@HA photosensitizer facilitated NIR photocatalytic oxygen hydrolysis, overcoming the hypoxia limitation in PDT. When combined with photothermal therapy, it displayed improved antitumor efficacy, offering a new strategy for the treatment of oral squamous cell carcinoma.

## Introduction

Oral cancer, encompassing malignancies originating within the oral cavity, persists as a major global health burden due to its aggressive biological behavior, elevated incidence rates, and unfavorable clinical outcomes. According to the International Agency for Research on Cancer, the incidence and mortality rates of oral cancer worldwide have been rising steadily each year (Table S1) [1–3]. In 2022, there were a total of 389,485 new cases of oral cancer reported worldwide, positioning it as the 16th most prevalent cancer. Additionally, there were 188,230 fatalities attributed to this disease, ranking it as the 15th leading cause of cancer-related deaths globally [2]. Oral cancer represents a specific subtype of head and neck tumors, with more than 90% of these cases being histologically categorized as oral squamous cell carcinoma (OSCC). The prevention and treatment of OSCC face severe challenges. Currently, the primary treatment approaches involve surgical resection in combination with chemotherapy and radiotherapy. Despite these interventions, the overall 5-year survival rate remains below 50% [4]. Furthermore, surgical resection can result in tissue defects, functional impairments, and even psychological disorders, markedly affecting the patients’ quality of life. Therefore, there is an urgent need to develop more effective treatment methods to improve both therapeutic effects and the overall well-being of patients.

Photodynamic therapy (PDT) is a highly promising cancer treatment, especially for superficial tumors, including skin cancer and oral cancer. PDT offers various advantages, including spatiotemporal selectivity, minimally invasive treatment, negligible drug resistance, and minimal toxicity [5]. PDT is particularly effective for OSCC, as it preserves surrounding tissues and aesthetics. Furthermore, it serves as the most effective palliative treatment for large or inoperable tumors [6]. PDT is classified into type I and type II PDT, with type II being the primary form. Type II PDT requires an oxygen-rich environment to be effective. However, the rapid proliferation of tumors reduces oxygen, causing hypoxia within the tumor, which is a common characteristic of the tumor microenvironment [7]. Hypoxia can activate hypoxia-inducible factor (HIF)-dependent signaling pathways, leading to immune suppression and resistance to apoptosis, promoting tumor cell proliferation, and contributing to tumor invasion and metastasis [8]. Thus, tumor hypoxia represents a major barrier to the effectiveness of PDT.

To address this challenge, researchers have developed various PDT strategies for hypoxic tumors, primarily focusing on 3 distinct aspects [9–11]: (a) directly or indirectly increasing the O_2_ concentration within the tumor, such as using photosensitizers in combination with hemoglobin or perfluorocarbons for O_2_ delivery, or generating in situ O_2_ using peroxidases or MnO_2_; (b) developing non-O_2_-dependent PDT strategies, such as type I PDT or hypoxia-sensitive photosensitizers; and (c) combining PDT with other non-O_2_-dependent treatments, such as photothermal therapy (PTT), hypoxia-activated anticancer drugs, or immunotherapy. However, these treatment strategies are still in the early stages of research and are complex to implement. Hence, there is a critical need for the development of more efficient and sustained oxygen supply methods to improve the efficacy of PDT. The development of inorganic nanophotosensitizers has emerged as an optimal approach due to their ease of modification, making them versatile photosensitizers for both PDT and PTT. PTT can moderately increase the local temperature of the tumor, improving blood circulation and thereby increasing local O_2_ levels, which enhances the effectiveness of PDT. Meanwhile, PTT is a non-O_2_-dependent treatment modality, and its combination with PDT can markedly enhance the overall therapeutic effect [12]. Further, inspired by photocatalytic water oxidation, water is considered a potential source for O_2_ production owing to its abundance as an endogenous substance in biological systems [13]. Therefore, this study developed inorganic nanophotosensitizers capable of near-infrared (NIR) activation and photocatalytic water hydrolysis, enabling the combined application of PDT and PTT for tumor treatment.

Bismuth vanadate (BiVO_4_) is an n-type semiconductor with a moderate bandgap (2.3 to 2.5 eV), a suitable valence band (VB) potential (higher than the 1.23 eV of H_2_O/O_2_), high photochemical stability, a low cost, and low toxicity, all of which make it a highly promising photocatalyst for the oxygen evolution reaction (OER); its typical crystal structure is monoclinic scheelite [14]. However, pure BiVO_4_ suffers from low photocatalytic water oxidation performance due to the easy recombination of electron–hole pairs. To effectively overcome this limitation, several modification strategies have been developed, including doping, forming heterojunction composites, and loading cocatalysts. For example, Ge et al. [15] integrated ultrathin (1.8-nm) FeOOH nanosheets (cocatalyst) with BiVO_4_ via electrostatic adsorption, resulting in an O_2_ release rate that was twice as high as that of pure BiVO_4_ under visible light photocatalysis. Wei et al. [16] synthesized ZnO/BiVO_4_ Z-type heterojunctions by employing hydrothermal methods, demonstrating that the O_2_ release rate of the ZnO/BiVO_4_ composite was significantly higher than that of pure BiVO_4_ under visible light. Yin et al. [17] prepared a novel butterfly-wing-shaped WO_3_/BiVO_4_ heterojunction using a one-step sol–gel method for photocatalytic water splitting. They reported that the O_2_ release after 5 h of light irradiation was 7.6 times higher than that of pure BiVO_4_. In summary, modified BiVO_4_ demonstrated outstanding performance in photocatalytic water splitting and O_2_ evolution.

Molybdenum disulfide (MoS_2_) is a 2-dimensional semiconductor nanomaterial having characteristics of both 2-dimensional materials and semiconductors. As a cocatalyst, MoS_2_ can modify semiconductor photocatalysts by effectively suppressing electron–hole pair recombination while increasing the number of catalytic sites, thereby considerably enhancing the photocatalytic performance of the semiconductors [18]. Li et al. [19] synthesized a well-structured MoS_2_/γ-Fe_2_O_3_/graphene ternary heterojunction via high-temperature calcination, which demonstrated a photocatalytic O_2_ release activity twice that of γ-Fe_2_O_3_/graphene under visible light irradiation. Further research has led to the synthesis of a SnS/MoS_2_ Z-type heterojunction, revealing that the unsaturated S atoms at the edges of the MoS_2_ nanoribbons can provide active sites for the OER [20]. Moreover, it was first confirmed in 2013 that MoS_2_ is an efficient NIR photothermal agent [21], demonstrating outstanding biocompatibility and widespread application in PTT for tumor treatment. Thus, it is postulated that combining MoS_2_ with BiVO_4_ can not only enhance the photocatalytic OER activity of BiVO_4_ but also extend the absorption spectrum into the NIR range, imparting photothermal properties to the composite material, thereby alleviating tumor hypoxia and improving tumor treatment efficacy.

**Fig. 1.**
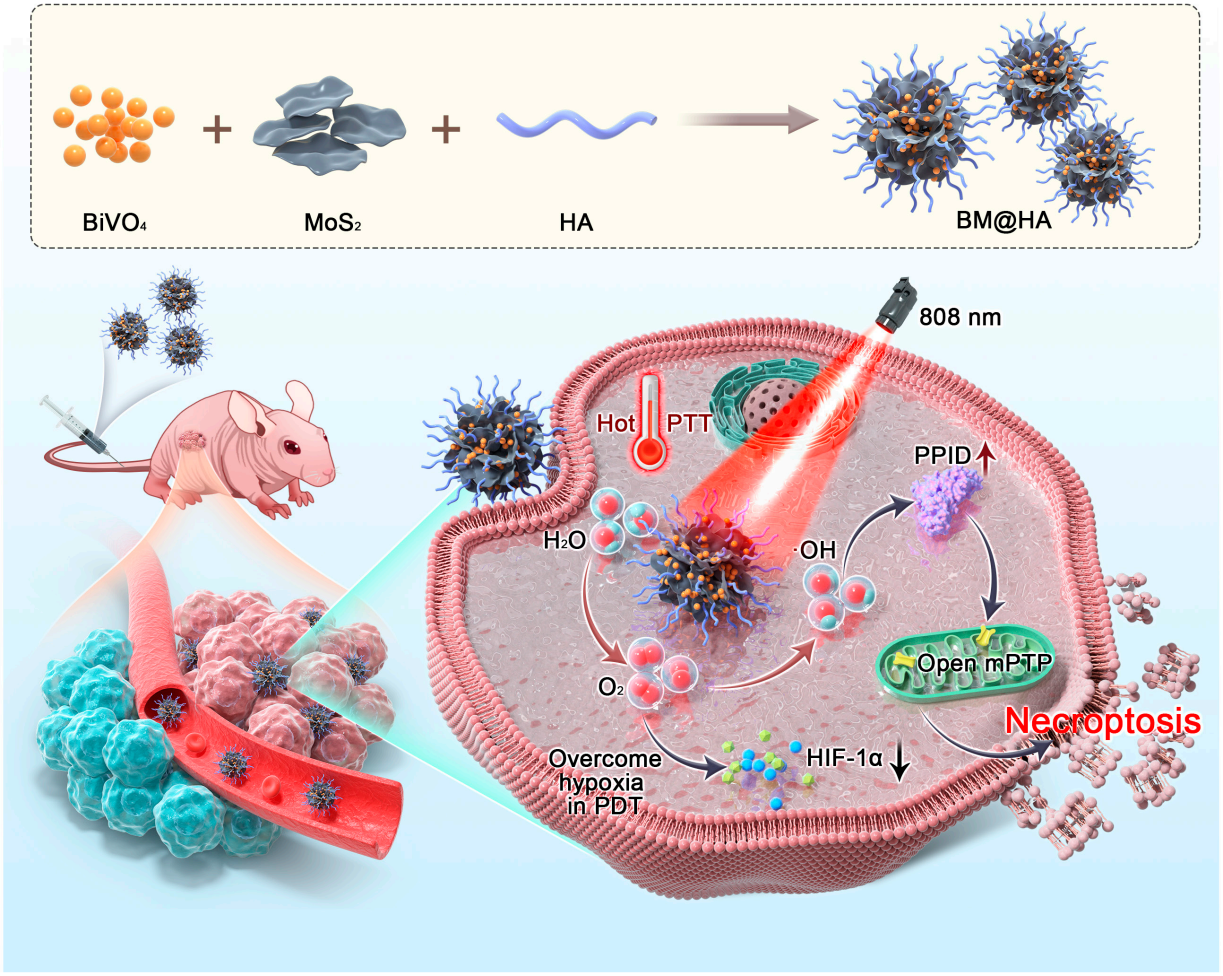
Schematic illustration of BiVO_4_/MoS_2_@hyaluronic acid (BM@HA) photosensitive particles utilized in hypoxic tumor therapy. Upon targeting the tumor, these nanoparticles exert therapeutic effects through 3 primary mechanisms: (a) Photodynamic therapy (PDT) stimulates the production of a substantial amount of reactive oxygen species (ROS; ·OH), which promotes an increase in peptidylprolyl isomerase D (PPID) protein levels, leads to the opening of the mitochondrial permeability transition pore (mPTP), and subsequently induces necroptosis in cells. (b) The photocatalytic decomposition of water produces O_2_, thereby enhancing the efficacy of PDT. (c) Photothermal therapy (PTT) directly induces cell death. HIF-1α, hypoxia-inducible factor 1-alpha.

In this study, a BiVO_4_/MoS_2_@hyaluronic acid (HA) (BM@HA) nanocomposite system with tumor-targeting features was synthesized, combining the advantages of 3 materials: (a) The photocatalytic water splitting and oxygen production characteristics of BiVO_4_ are anticipated to utilize the abundant water in living organisms. (b) The combination of MoS_2_ with BiVO_4_ can markedly enhance the photocatalytic performance of BiVO_4_. (c) MoS_2_ endows the composite material with outstanding photothermal performance and NIR absorption potential, while a moderate temperature increase can accelerate tumor blood flow and alleviate hypoxia. (d) HA provides the system with tumor-targeting functionality. After irradiation with an 808-nm laser, the effects of BM@HA on water oxidation and its combined PDT and PTT therapeutic effects on OSCC were evaluated, offering new strategies for targeted and efficient treatment of this cancer (Fig. 1).

## Materials and Methods

### Molecular dynamics simulations

Initially, a force field was constructed using a machine learning-based approach in combination with the Vienna Ab initio Simulation Package (VASP) based on first principles. Molecular dynamics simulations were then performed using VASP. The calculation accuracy was set to “Accurate”. The parameters were defined as EDIFF = 1 × 10^−4^ eV and EDIFFG = −1 × 10^−2^ eV/Å, and the temperature remained constant at 300 K. A minimum vacuum thickness of 30 Å was maintained in all cases to avoid interactions between adjacent layers. The binding energy *E*_b_ was defined as the total energy of the composite system minus the total energies of each material, such as *E*_b(BM@HA)_ = *E*_tot_ − *E*_(MoS2)_ − *E*_(BiVO4)_ − *E*_(HA)_.

### Synthesis of BiVO_4_ nanoparticles

To a 250-ml 3-neck flask, 2 mmol of Bi(NO_3_)_3_·5H_2_O, 20 ml of octadecene, 4 ml of oleic acid, and 4 ml of oleylamine were added. The mixture was vigorously stirred under a nitrogen atmosphere and heated to 175 °C. Once the solution became completely cleared, the heating mantle was removed, allowing the solution to naturally cool to 130 °C. After that, the vanadium precursor solution (4 mmol of NH_4_VO_3_ dissolved in 20 ml of boiling water with stirring to ensure complete dissolution) was quickly added to the mixture. The solution was then stirred vigorously and maintained at 100 °C for 5 min and then naturally cooled to room temperature, and the product was purified. Next, 20 ml of ethanol was added to the reaction mixture, and the mixture was thoroughly mixed. After allowing the mixture to settle, the lower aqueous layer was discarded. The upper organic phase was then treated with water (20 ml) followed by an additional 20 ml of ethanol, ensuring thorough mixing. After standing, the lower aqueous layer was removed again and the process was repeated twice. Finally, the product was purified by washing 3 times with ethanol at 5,000 rpm for 5 min and freeze-dried to yield the final product.

### Synthesis of MoS_2_ nanosheets

Ammonium molybdate (2 mmol) and 30 mmol of thiourea were dissolved in 38 ml of deionized water, and the mixture was stirred for 10 min, followed by sonication for another 10 min. The mixture was placed in a polytetrafluoroethylene autoclave and heated at 180 °C for 24 h. After allowing it to cool to room temperature, the product was purified through centrifugation with deionized water at 5,000 rpm for 5 min, repeated 3 times. It was then centrifuged with ethanol under the same conditions thrice and finally freeze-dried.

### Synthesis of BM@HA

Initially, methyl methacrylate-modified HA (m-HA) was synthesized. Briefly, 1.0 g of HA was dispersed in 50 ml of deionized water and allowed to swell at 4 °C overnight, which was then added with 0.8 ml of methacrylic anhydride, and the pH of the solution was adjusted to 8 to 9 using a 5 M NaOH solution. The reaction mixture was then stirred continuously at 4 °C for 24 h to ensure complete modification. Following this, the product was precipitated in acetone, and the resulting solid was filtered, washed with ethanol, and air-dried.

Secondly, 15 mg of m-HA was completely dissolved in 100 ml of deionized water and added with 100 μl of NaOH (0.1 M) and 500 mg of BiVO_4_ and MoS_2_ (in different mass ratios). The mixture was then subjected to ultrasonication at 4 °C for 30 min to ensure homogeneous dispersion of the components. After that, the cross-linking agent *N*,*N*′-methylenebisacrylamide (15 mg) and the photoinitiator Irgacure 2959 (0.1%, W:V) were added to the above prepared dispersion. The polymerization of m-HA was initiated under ultraviolet (UV) irradiation for 5 min, and the resulting product was separated by centrifugation followed by purification with deionized water.

### Characterization of materials

The morphology and size of the developed systems were analyzed using transmission electron microscopy (TEM; Tecnai F30). The hydrodynamic size and zeta potential of the samples was obtained using a Zetapals analyzer. The composition of the samples was determined by Fourier transform infrared spectroscopy (FTIR) using a Thermo Nicolet Nexus 670 attenuated total reflectance–infrared spectrometer, while UV absorption spectra were recorded using a Genesys 10S UV–visible (UV–Vis) spectrophotometer. X-ray diffraction (XRD) patterns were obtained using a Rigaku Ultima IV diffractometer. X-ray photoelectron spectroscopy (XPS) analysis was performed using a VG ESCALAB 220I-XL spectrometer. Fluorescence spectra were recorded using a HORIBA FL-3 system. The data were processed using the Origin software for accurate replotting and interpretation.

### Photothermal performance of BM@HA

BM@HA with different concentrations (0, 25, 50, 100, and 200 μg ml^−1^) was irradiated with an 808-nm laser at various power densities (0.5, 0.75, 1.0, and 1.5 W cm^−2^) for 10 min. The solution temperature was monitored using a German infrared thermal imager (testo 865), capturing images at 60-s intervals. The photothermal conversion efficiency of BM@HA was then calculated according to the following formulas:

$$\eta =\frac{\mathrm{hs}\left(T_{\max}-T_{\text{surr}}\right)-Q_{0}}{I\left(1-10^{-A_{808}}\right)}$$ (1)

$$\;\tau_{s}=\frac{m_{d}C_{d}}{\mathrm{hs}}$$ (2)

$$\;Q_{0}=\mathrm{hs}\left(T_{\max,\text{water}}-T_{\text{surr}}\right)$$ (3)

### Detection of ROS and O_2_ generation

Reactive oxygen species (ROS) generation was detected using 2′,7′-dichlorodihydrofluorescein diacetate (DCFH-DA). DCFH-DA solution (3 μl) was added to a 3-ml dispersion containing various samples (100 μg ml^−1^, phosphate-buffered saline [PBS] pH 7.4). The mixture was irradiated with an 808-nm laser (0.75 W cm^−2^) for 5 min, followed by incubation at 37 °C for 2 h. After centrifugation, the supernatant was collected and analyzed using fluorescence spectroscopy.

O_2_ generation was measured using a dissolved oxygen meter (JPBJ-608, China). A mixture of PBS solution containing 0.03 mol l^−1^ AgNO_3_ and the composite material was purged with N_2_ and sealed with liquid paraffin. After NIR irradiation with an 808-nm laser (0.75 W cm^−2^) for 10 min, oxygen levels were measured using the dissolved oxygen meter.

### In vitro cytotoxicity evaluation

L929 cells were seeded into 96-well plates at a density of 5,000 cells/well and cultured at 37 °C and 5% CO_2_ for 12 h. Various concentrations of 25BM@HA ranging from 0 to 200 μg ml^−1^ were then introduced, followed by an additional 24-h incubation. Cell viability was determined using the standard Cell Counting Kit-8 (CCK-8) method with absorbance recorded at 450 nm with a microplate reader.

Biocompatibility was further evaluated through a hemolysis analysis. Mouse blood (0.5 ml) was centrifuged and washed 5 times with PBS to obtain erythrocytes, which were then diluted 10-fold in PBS. The diluted suspension (200 μl) was mixed with 800 μl of varying concentrations of 25BM@HA PBS solution (0 to 400 μg ml^−1^) or Triton X-100 (0.025% in PBS) and incubated at 37 °C for 2 h. After centrifugation, the absorbance of the supernatant was measured at 541 nm to assess hemolysis.

### Detection of the cellular uptake of BM@HA

Fluorescein isothiocyanate (FITC)-labeled BM@HA was used for cellular uptake detection. L929, Cal-27, SAS, and SCC9 cells were seeded at a density of 2.0 × 10^4^ cells/well into 24-well plates and cultured overnight. The cells were then treated with 25BM@HA (fluorescently labeled) for 6 h. After washing with PBS 3 times, the cells were observed under a microscope and analyzed by flow cytometry.

### The effect of BM@HA on tumor cell proliferation

Cal-27 and SAS cells were seeded into 96-well plates at a density of 5,000 cells per well and cultured overnight. The cells were treated with varying concentrations of 25BM@HA (0 to 50 μg ml^−1^) for 6 h, followed by NIR laser irradiation (808 nm, 0.75 W cm^−2^) for 5 min. After an 18-h incubation, cell viability was evaluated using the CCK-8 assay by recording the absorbance at 450 nm to identify the most effective concentration for further analysis. The hypoxia experiments were conducted in a tri-gas incubator (1% O_2_, 5% CO_2_, 37 °C).

Live-cell staining using calcein-AM was conducted to evaluate the impact of 25BM@HA on the viability of tumor cells. Cal-27 and SAS cells were seeded at a density of 2.0 × 10^4^ cells per well in a 24-well plate. After 12 h of incubation, the cells were treated with or without 25BM@HA (50 μg ml^−1^) for 6 h, followed by 5 min of irradiation with an 808-nm laser (0.75 W cm^−2^) or no irradiation. The cells were assigned to 4 groups: control group, NIR group, 25BM group, and 25BM + NIR group. After 18 h of continued incubation, the culture medium was removed, and the cells were washed twice with PBS followed by the addition of 0.5 ml of diluted (1:1,000) calcein-AM working solution. The cells were then incubated in the dark at 37 °C for 30 min and observed under a fluorescence microscope.

Finally, the colony formation assay was performed to evaluate the effect of 25BM@HA on tumor cell proliferation. Cal-27 and SAS cells were seeded at a density of 500 cells per well into a 24-well plate. Subsequently, the cells were treated with 25BM@HA for 6 h or left untreated, followed by either 5 min of 808-nm laser irradiation (0.75 W cm^−2^) or no irradiation. The experimental setup consisted of 4 groups: control, NIR group, 25BM group, and 25BM + NIR group. Following an 18-h incubation, the culture medium was removed, and the cells were washed 3 times with PBS before adding fresh culture medium for a further 10 d. After fixing the cells with 0.5 ml of methanol in the dark for 15 min, methanol was removed, followed by staining with 0.5 ml of Giemsa stain for 30 min. The cells were washed with flowing water, dried, and photographed using a camera. The number of colonies with more than 50 cells was counted using the ImageJ software to calculate the numbers of colonies.

### Evaluation of intracellular ROS and O_2_ levels

Intracellular ROS and O_2_ levels were assessed using DCFH-DA and tris(4,7-diphenyl-1,10-phenanthroline)ruthenium(II) dichloride ([Ru(dpp)_3_]Cl_2_) probes, respectively. Cal-27 and SAS cells were seeded at a density of 2.0 × 10^4^ cells per well into a 24-well plate overnight. Afterward, the cells were treated with 25BM@HA for 6 h and exposed to NIR laser irradiation (808 nm, 0.75 W cm^−2^) for 5 min. After 2 h of incubation, the cells were washed 3 times with PBS, and the corresponding reagents were added according to the kit protocols. The cells were washed 3 times with PBS, photographed under a fluorescence microscope, and analyzed by flow cytometry.

### Western blot

SAS cells were treated with or without 25BM@HA for 6 h, followed by 5 min of irradiation with an 808-nm laser (0.75 W cm^−2^) or no irradiation. The cells were categorized into 4 groups: control group, NIR group, 25BM group, and 25BM + NIR group. Following further incubation for 2 h, proteins were extracted using radioimmunoprecipitation assay lysis buffer (Thermo Fisher Scientific) and quantified with a bicinchoninic acid assay kit (Beyotime Biotechnology). Protein samples were separated by 10% or 12% sodium dodecyl sulfate–polyacrylamide gel electrophoresis and transferred to polyvinylidene fluoride membranes using a semidry transfer apparatus. The following primary antibodies were used: HIF-1α (1:1,000, Servicebio, China), Nrf2 (1:500, Huabio, China), HO-1 (1:1,000, Immunoway, USA), peptidylprolyl isomerase D (PPID; 1:500, Proteintech, China), RIPK1 (1:1,000, Immunoway, USA), RIPK3 (1:1,000, Abcam, USA), MLKL (1:1,000, Immunoway, USA), and β-actin (1:5,000, Immunoway, USA). Protein bands were detected using enhanced chemiluminescence detection reagents.

### Evaluation of in vivo tumor phototherapy

Twenty-five male nude mice (4 to 6 weeks old) were purchased from SPF (Beijing) Biotechnology Co., Ltd., and randomly divided into 5 groups (*n* = 5): control group, NIR group, 25BM@HA group, and 25BM@HA + NIR group. Five additional mice were used to assess the biodistribution and photothermal imaging of 25BM@HA in vivo. After 1 week of the acclimatization period, SAS cells (5.0 × 10^6^/200 μl) were injected into the right forelimb axilla of the mice to establish tumors.

When the tumor size reached 100 to 150 mm^3^, in vivo thermal imaging of 25BM@HA was conducted using a thermal infrared imager. Mice were intravenously injected with PBS (150 μl) containing 25BM@HA (10 mg kg^−1^), followed by irradiation with an 808-nm laser for 5 min at 0, 1, 6, 12, and 24 h postinjection. Thermal images and recordings were taken at 60-s intervals, providing real-time monitoring of the temperature changes. In vivo imaging was used to detect the biodistribution of 25BM@HA. Following intravenous injection of fluorescently labeled 25BM@HA (10 mg kg^−1^), the mice were anesthetized at 6, 12, and 24 h. The distribution of 25BM@HA in nude mice was then observed using a versatile imaging system (Bio-Rad ChemiDoc MP). After euthanizing the mice, heart, liver, spleen, lung, kidney, and tumor tissues were harvested for imaging using the versatile imaging system. Fluorescence intensity analysis was subsequently performed using the ImageJ software.

Treatment was started when the tumor size reached approximately 80 mm^3^ (day 0), with mice receiving intravenous injections of 150 μl of PBS, either with or without 25BM@HA (10 mg kg^−1^). After 12 h, the mice were treated with an 808-nm laser (0.75 W cm^−2^, 5 min) irradiation or no irradiation. Treatment was administered every 2 d, with the tumor size and mouse weight monitored throughout the study. After 2 weeks of treatment, the mice were euthanized, and major organs (heart, liver, spleen, lung, and kidney) along with tumor tissues were harvested for histological analysis using hematoxylin and eosin (HE) staining. Immunofluorescence was performed to detect HIF-1α expression in tumors, while immunohistochemistry (IHC) was used to assess Ki-67 expression in tumor tissues.

All animal experiments were conducted following the “Guide for the Care and Use of Laboratory Animals” by the National Institutes of Health and were approved by the Medical Ethics Committee of the School of Stomatology, Lanzhou University (LZUKQ-2024-038).

### Proteomic analysis

Tumor proteomics were evaluated by analyzing tissues derived from treated nude mice, with 3 samples belonging to each of the control and 25BM@HA + NIR groups. The tissues were rapidly frozen in liquid nitrogen and submitted to Shanghai Genechem Co., Ltd. for proteomic analysis to identify differentially expressed proteins. Gene Ontology (GO) annotation of the target protein set was performed using Blast2GO (V1.4.4), with the GO database version go.obo (2019.07.01). For Kyoto Encyclopedia of Genes and Genomes (KEGG) pathway annotation, the KOALA (KEGG Orthology and Links Annotation, V3.0) software was employed to perform comparisons against the KEGG GENES database. The target protein sequences underwent KO classification, and pathway information associated with these sequences was automatically obtained according to KO classification. The KEGG database version number was KO_INFO_END.txt (2024.06.03). Furthermore, Fisher’s exact test was applied to compare the distribution of each GO term or KEGG pathway between the target protein set and the overall protein set, determining the significance level of the enrichment of a specific GO term or KEGG pathway. Moreover, interaction networks, with direct or indirect relationships to the target proteins, were searched in the String database using the target protein ID. The resulting interaction network analysis was visualized using the AnyChart software (V8.11.0.1934).

### Statistical analysis

All experiments were repeated 3 times, and data were analyzed using *t* tests through the SPSS software. All data are presented as mean ± SD. A *P* value of <0.05 indicates a statistically significant difference: *P < 0.05, ** *P* < 0.01, and *** *P* < 0.001.

## Results and Discussion

### Synthesis and characterization of BM@HA

**Fig. 2.**
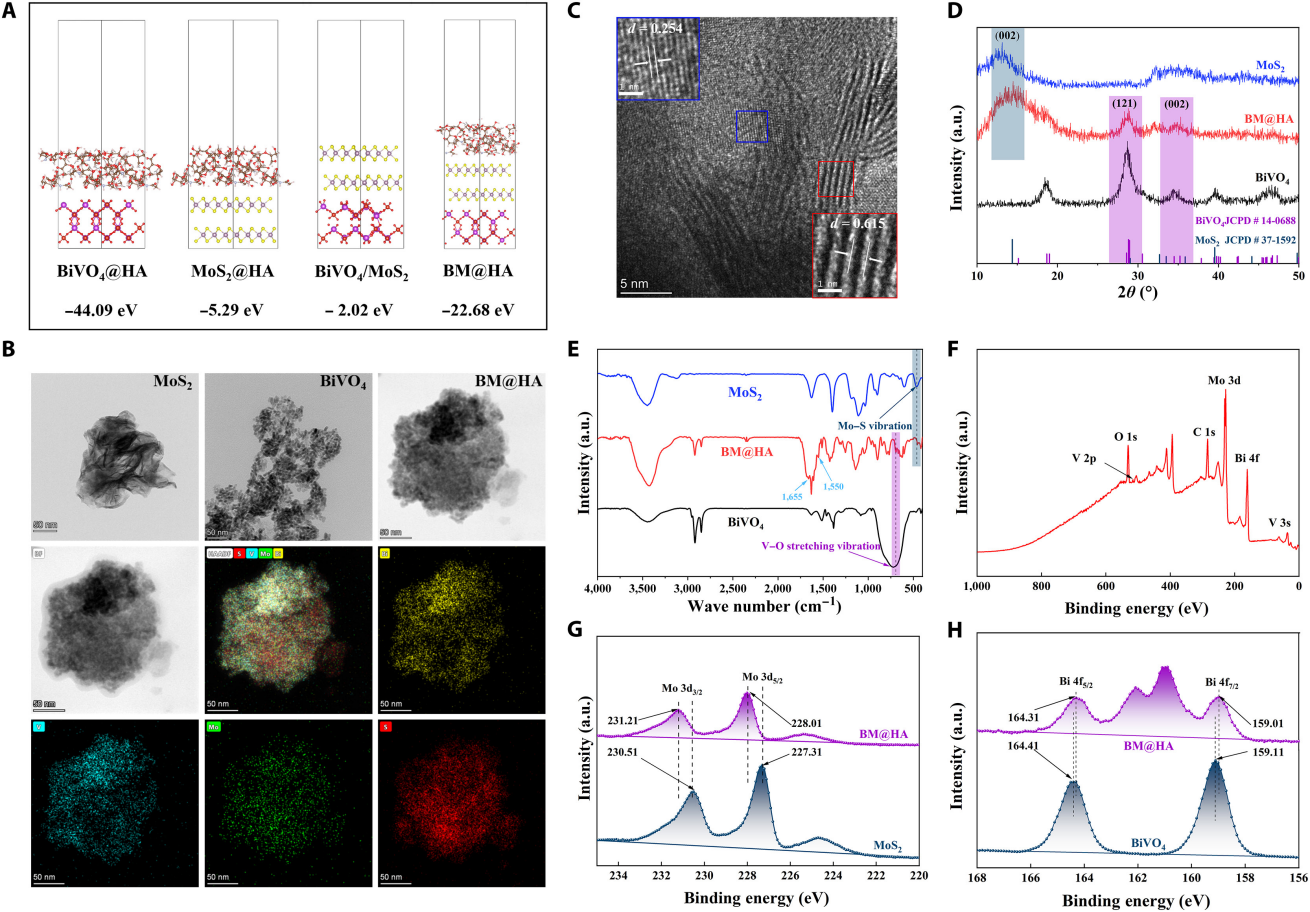
Characterization of BM@HA. (A) Molecular dynamics simulations of BiVO_4_@HA, MoS_2_@HA, and BM@HA. (B) Transmission electron microscopy (TEM) of BiVO_4_, MoS_2_, and BM@HA and corresponding elementary mapping of BM@HA (scale bar = 50 nm). (C) High-resolution TEM (HRTEM) of BM@HA (scale bar = 5 nm, scale bar of the magnified image = 1 nm). (D) X-ray diffraction (XRD) patterns of BiVO_4_, MoS_2_, and BM@HA. (E) Fourier transform infrared spectroscopy (FTIR) of BM@HA. (F) The survey x-ray photoelectron spectroscopy (XPS) spectrum of BM@HA. (G) XPS spectra of Mo 3d in BM@HA and MoS_2_. (H) XPS spectra of Bi 4f in BM@HA and BiVO_4_.

Molecular dynamics simulations were conducted using VASP to evaluate the feasibility and stability of the composite system comprising MoS_2_, BiVO_4_, and HA. These simulations calculated the interaction forces between the 3 components (Fig. 2A). The binding energies of BiVO_4_@HA, MoS_2_@HA, and BM@HA were determined to be −44.09, −5.29, and −22.68 eV, respectively. All values were negative, indicating that the 3 synthesized systems were relatively stable. Moreover, more negative binding energy correlates with enhanced stability, providing a theoretical basis for the follow-up experiments.

MoS_2_ and BiVO_4_ nanoparticles were synthesized via hydrothermal and solvothermal methods, respectively, and characterized using TEM, XRD, and FTIR. TEM results showed that MoS_2_ formed thin-layer nanosheets, while BiVO_4_ appeared as nanoparticles with diameters smaller than 10 nm (Fig. 2B and Fig. S1A, B, D, and E). In pure BiVO_4_, a lattice fringe of 0.310 nm was observed (Fig. S1C), corresponding to the (121) phase of monoclinic BiVO_4_ (JCPDS no. 14-0688). Pure MoS_2_ showed a lattice fringe of 0.615 nm (Fig. S1F), corresponding to the (002) plane of 2H MoS_2_ (JCPDS no. 371492), which was consistent with the XRD results (Fig. 2D). Further FTIR analysis revealed that there was a typical Mo–S stretching vibration peak at 436 cm^−1^ for MoS_2_ [22], while the absorption band appeared in the range of 700 to 900 cm^−1^ was attributed to the asymmetric stretching of the V–O bond present in BiVO_4_ (Fig. 2E) [23]. MoS_2_ and BiVO_4_ were combined using a simple method and modified with low-molecular-weight HA (20,000 Da) to synthesize BM@HA. TEM results revealed that the final synthesized BM@HA displayed a morphology similar to that of MoS_2_ nanosheets, with BiVO_4_ nanoparticles attached to their surfaces (Fig. 2B and Fig. S1G). The hydrodynamic diameter of BM@HA was measured to be 217.34 ± 12.23 nm (Fig. S1H). The zeta potential of BM@HA was recorded at −19.7 ± 5.01 mV (Fig. S1H). Upon dispersion in water, no marked agglomeration was observed after 24 h (Fig. S1I), and a very small amount of aggregation was observed after 7 d. Upon dispersion in PBS, Dulbecco’s modified Eagle medium, and complete medium, similar results were obtained. However, BiVO_4_/MoS_2_ was dispersed in water, and substantial aggregation was observed after 7 d (Fig. S1J). The results indicated that BM@HA exhibits excellent dispersibility and HA could improve the dispersion of BiVO_4_/MoS_2_. High-resolution TEM images of BM@HA revealed 2 lattice fringes with spacings of 0.615 and 0.254 nm, corresponding to the (002) planes of MoS_2_ and BiVO_4_, respectively (Fig. 2C). The XRD results indicated that BM@HA corresponds to the (002) and (121) planes of BiVO_4_, as well as the (002) plane of MoS_2_ (Fig. 2D). Meanwhile, BM@HA nanoparticles demonstrated a translucent outer layer, attributed to the HA layer grafted on the surface (Fig. 2B). The elemental mapping images from TEM revealed an even distribution of Bi, V, Mo, and S in BM@HA, indicating that BiVO_4_ was adhered to the surface of MoS_2_ nanosheets, contributing to the formation of a 3-dimensional structure. These results confirmed the successful synthesis of BM@HA. Moreover, the FTIR spectrum of BM@HA displayed a characteristic peak of Mo–S stretching vibration at 436 cm^−1^ and a V–O asymmetric stretching in the 700 to 900 cm^−1^ range. Meanwhile, the bands of BM@HA at 1,655 and 1,550 cm^−1^ could be assigned to amide I and amide II, attributed to the carbonyl stretching and N–H bending of HA, respectively [24,25]. These results further validated the successful synthesis of BM@HA.

XPS was employed to further investigate the formation and valence states of the BM@HA nanocomposite. The analysis displayed the presence of Bi, V, S, and Mo, as well as C and O, which may primarily originate from organic modification (Fig. 2F). In the Bi 4f XPS spectrum of BiVO_4_ (Fig. 2H), binding energies of 159.11 and 164.41 eV were assigned to Bi 4f_7/2_ and Bi 4f_5/2_, respectively, with a difference of 5.3 eV, which was consistent with the presence of Bi^3+^ [26]. In the Mo 3d XPS spectrum of MoS_2_ (Fig. 2G), binding energies of 230.51 and 227.31 eV were assigned to Mo 3d_3/2_ and Mo 3d_5/2_, respectively, with a binding energy difference of 3.2 eV, consistent with Mo^6+^ [27]. On the other hand, after the combination of MoS_2_ and BiVO_4_, the binding energies of Mo 3d_3/2_ and Mo 3d_5/2_ increased, while those associated with Bi 4f_7/2_ and Bi 4f_5/2_ showed a prominent decrease (Fig. 2G and H). The shift in binding energies indicated the process of electron transfer from MoS_2_ to BiVO_4_ in the BM@HA composite.

### Optical properties of BM@HA

**Fig. 3.**
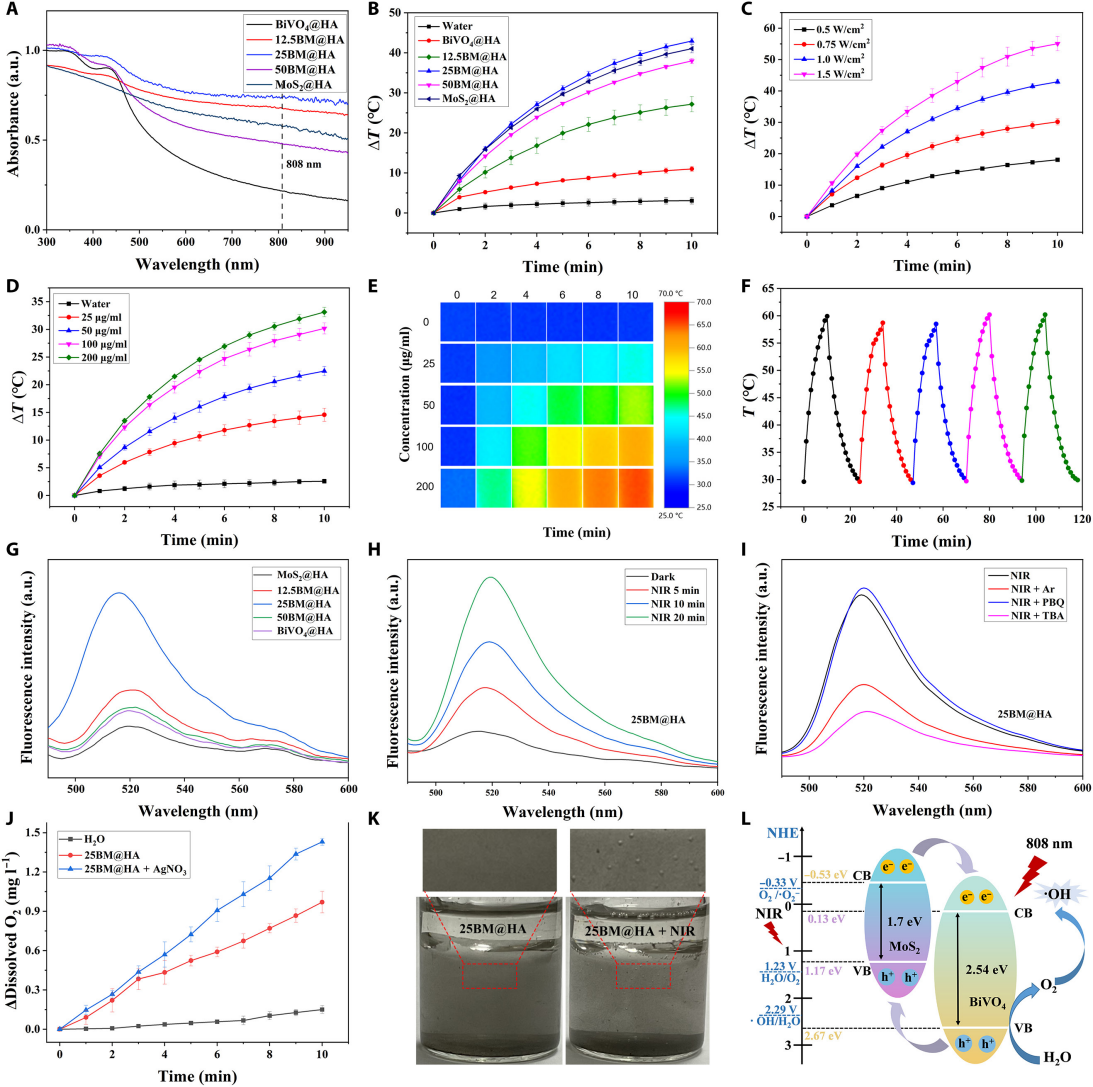
Optical properties of BM@HA. (A) Ultraviolet–visible (UV–Vis) absorption spectra of the samples. (B) Photothermal heating curves of the aqueous dispersions of these samples (100 μg ml^−1^) under 808-nm laser irradiation (1 W cm^−2^). (C) Photothermal heating curves of the 25BM@HA suspension at different laser powers (808 nm). (D) Photothermal heating curves of the aqueous dispersions of 25BM@HA at different concentrations under 808-nm laser irradiation (0.75 W cm^−2^). (E) Infrared thermal images of the aqueous dispersions of 25BM@HA at different concentrations and irradiation times. (F) Photothermal recycle curve of 25BM@HA. (G) Fluorescence spectra of dichlorofluorescein diacetate (DCFH-DA) treated by different samples (100 μg ml^−1^) under near-infrared (NIR) irradiation (808 nm, 0.75 W cm^−2^). (H and I) Fluorescence spectra of DCFH-DA and 25BM@HA solution under different conditions. (J) O_2_ generation curves of 25BM@HA with or without AgNO_3_. (K) O_2_ generation photographic images of 25BM@HA (without AgNO_3_). (L) Schematic illustration of the photocatalytic mechanism of BiVO_4_/MoS_2_ for generating O_2_ and ROS. PBQ, *p*-benzoquinone; TBA, *tert*-butanol; NHE, normal hydrogen electrode; CB, conduction band; VB, valence band.

The MoS_2_@HA, 12.5BM@HA, 25BM@HA, 50BM@HA, and BiVO_4_@HA composites were prepared by adjusting the BiVO_4_ content to 0%, 12.5%, 25%, 50%, and 100%, respectively. The UV–Vis absorption spectra revealed that all samples showed prominent absorption in the NIR region, as shown in Fig. 3A. At lower BiVO_4_ doping levels, both 12.5BM@HA and 25BM@HA showed enhanced absorption in the NIR region compared to MoS_2_@HA, with the absorption intensity increasing as the BiVO_4_ content increased. The observed enhancement in absorption occurred because doping introduced new energy levels within the semiconductor bandgap, thereby enhancing light absorption. However, when the BiVO_4_ content reached 50%, the NIR absorption of 50BM@HA was found to be lower than that of MoS_2_@HA, suggesting that optimal doping levels are essential for maximizing absorption. To investigate the photothermal performance, samples were dispersed in water at a concentration of 100 μg ml^−1^ and exposed to an 808-nm NIR laser (1 W cm^−2^) for 10 min. Temperature changes were then monitored, as shown in Fig. 3B. All samples showed a temperature increase, aligning with the UV absorption analysis. Among them, 25BM@HA demonstrated the highest absorption in the NIR region (808 nm), resulting in the most substantial temperature rise, reaching up to 42.9 °C. Furthermore, the photothermal capability of 25BM@HA showed a positive correlation with both the laser irradiation intensity (Fig. 3C) and the sample concentration (Fig. 3D). Upon irradiation with an 808-nm NIR laser at a power density of 0.75 W cm^−2^ for 10 min, 25BM@HA demonstrated a concentration-dependent temperature increase, ranging from 14.5 °C at 25 μg ml^−1^ to 33.1 °C at 200 μg ml^−1^, in contrast to water, which showed a negligible rise of only 2.5 °C. These findings were further validated through thermal imaging (Fig. 3E). Moreover, after 5 cycles of laser irradiation, 25BM@HA maintained a consistent temperature rise without any sign of attenuation, highlighting its exceptional photothermal stability (Fig. 3F). Furthermore, the material demonstrated a high photothermal conversion efficiency of 51.9% (Fig. S2). These results demonstrate that 25BM@HA possesses outstanding photothermal tunability and stability, establishing its potential as a promising photothermal agent for PTT application.

Afterward, the potential of BM@HA for application in PDT was investigated. PDT is widely recognized for its mechanism of generating ROS upon exposure to specific light irradiation. In this study, DCFH-DA was used as a probe to assess the ROS generation capability after 808-nm (0.75 W cm^−2^) NIR laser irradiation via fluorescence spectroscopy. The oxidation of DCFH-DA by ROS produced 2′,7′-dichlorofluorescein, a compound that emits green fluorescence with an intensity directly proportional to the ROS concentration. As shown in Fig. 3G, all samples displayed marked fluorescence emission at 522 nm after laser irradiation, confirming ROS generation. The ROS production levels followed the order 25BM@HA > 12.5BM@HA > 50BM@HA > BiVO_4_@HA > MoS_2_@HA. 25BM@HA generated the most ROS after laser irradiation, and this was time dependent (Fig. 3H). Given the excellent photothermal performance and ROS generation capability of 25BM@HA, we selected 25BM@HA (or written as 25BM) for further experiments. To determine the types of ROS, *tert*-butanol and *p*-benzoquinone were used as ·OH and ·O_2_^−^ scavengers, respectively. The results revealed that *tert*-butanol markedly reduced fluorescence intensity, while *p*-benzoquinone had no marked effect (Fig. 3I), indicating that ·OH was the primary reactive species, rather than ·O_2_^−^. Moreover, the requirement for O_2_ in ROS generation was confirmed by the substantial decrease in fluorescence intensity when argon was bubbled through to remove dissolved O_2_ (Fig. 3I). The decomposition of water by 25BM@HA to yield O_2_ upon laser irradiation was examined by measuring the dissolved oxygen levels in water using a dissolved oxygen analyzer. Initially, the dissolved oxygen was removed with argon, and after 10 min of 808-nm laser irradiation, the dissolved oxygen levels increased by 1.4 mg l^−1^ in 25BM@HA + AgNO_3_ (electron capture agent), while a 1.0 mg l^−1^ increase was observed in the absence of AgNO_3_ (Fig. 3J). Moreover, the formation of numerous bubbles in the solution was observed (Fig. 3K), confirming that 25BM@HA could decompose water to generate O_2_ upon 808-nm laser irradiation. The described process replenished the O_2_ consumed during ROS generation, highlighting its potential for PDT therapy.

Finally, to investigate the mechanism of ROS and O_2_ generation, we conducted UV–Vis diffuse reflection spectroscopy on the samples. The results revealed a pronounced redshift in the spectrum of 25BM@HA, which could be excited by NIR light (Fig. S3A to C). According to the formula of (*αhν*)^1/*n*^ = *B*(*hν* − *E*_g_), we calculated the bandgap of the samples from the (*αhν*)^1/*n*^–*hν* curve. The findings indicated that the bandgaps of BiVO_4_, MoS_2_, and 25BM@HA were 2.54, 1.7, and 1.38 eV, respectively (Fig. S3D to F). UV photoelectron spectroscopy was employed to evaluate the VB of each sample; results showed that the VB values were 2.67 eV for BiVO_4_ and 1.17 eV for MoS_2_ (Fig. S4). It could be calculated from the above results (*E*_CB_ = *E*_VB_ − *E*_g_) that the conduction band (CB) of BiVO_4_ was 0.13 eV and the VB of MoS_2_ was −0.53 eV. Based on their respective band structures, BiVO_4_ and MoS_2_ could form an n–p heterojunction. Due to its smaller bandgap, MoS_2_ could first be excited under irradiation with an 808-nm laser irradiation; this excitation generated electrons (e^−^) in its CB, which were subsequently transferred to the CB of BiVO_4_. This transfer was corroborated by the results of XPS. Concurrently, owing to its photothermal effect, MoS_2_ enhanced thermal energy within the composite material, facilitating further excitation of BiVO_4_, and this process generated holes (h^+^) in its VB, which then migrate to the VB of MoS_2_. It has been observed that O_2_ can be produced through reactions between photoexcited holes and water molecules (2H_2_O + 4h^+^ → O_2_ + 4H^+^, >1.23 V vs. normal hydrogen electrode [NHE]) [28]; additionally, strongly oxidizing holes may directly react with water, resulting in ·OH (h^+^ + H_2_O → ·OH + H^+^, >2.29 V vs. NHE) [25]. Furthermore, electrons generated during this process can interact with O_2_, leading to ultra-oxygen free radical formation(O_2_ + e^−^ → ·O_2_^−^, <−0.33 V vs. NHE) [29]. Specifically, the mechanism of 25BM@HA stimulating the generation of O_2_ and ROS is shown in Fig. 3L. 25BM@HA (1.38 eV) with a narrow bandgap could generate active electrons and free holes in the CB and VB after 808-nm laser irradiation. Notably the VB of BiVO_4_ was positive enough (2.67 eV, >2.29 V vs. NHE), and the free holes in VB could react with water to produce O_2_ and ·OH. Moreover, the CB of MoS_2_ exhibited a sufficiently negative potential (−0.53 eV, <−0.33 V vs. NHE), enabling the free electrons in the CB to react with O_2_ and form ·O_2_^−^, and then combined with H^+^ to form ·OH. Therefore, the heterojunction 25BM@HA designed and synthesized in this study could react with water to generate O_2_ and ROS after NIR laser irradiation, overcoming tumor hypoxia and exhibiting strong PDT performance.

### The impact of 25BM@HA on OSCC cells

The biocompatibility of 25BM@HA was first assessed by evaluating the viability of L929 cells using the CCK-8 assay. The results showed that even at a concentration of 200 μg ml^−1^, the viability of L929 cells remained above 90% (Fig. S5A), indicating the favorable cellular compatibility of 25BM@HA. Furthermore, the blood compatibility of 25BM@HA was assessed using a hemolysis experiment carried out on mouse red blood cells (Fig. S5B). No noticeable hemolysis was observed at a 400 μg ml^−1^ concentration, suggesting that 25BM@HA has favorable blood compatibility.

**Fig. 4.**
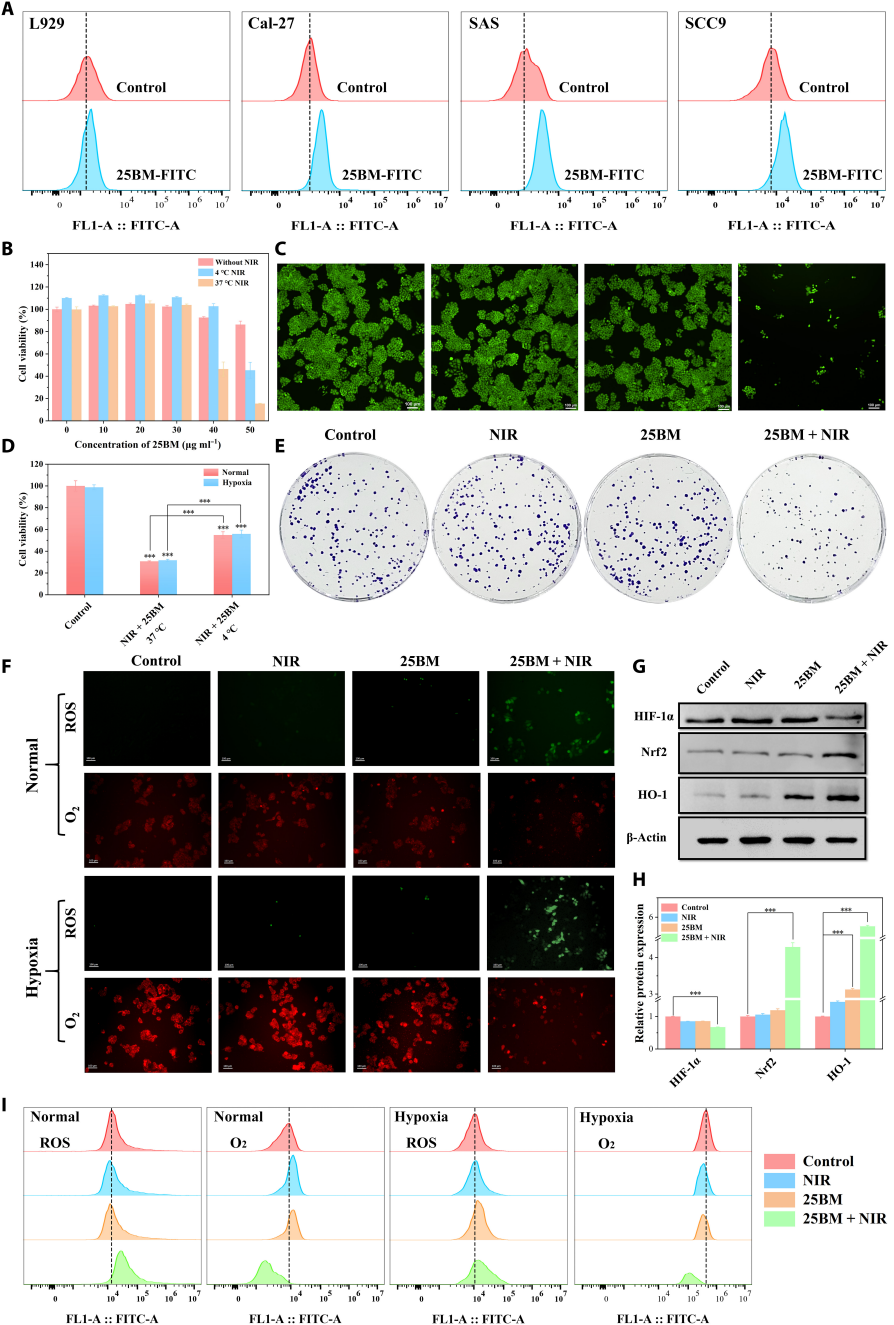
The effect of 25BM@HA on oral squamous cell carcinoma (OSCC) cells. (A) The cellular uptake behavior of 25BM@HA after incubation for 6 h was determined by flow cytometry. (B) Viability of Cal-27 cells assessed by Cell Counting Kit-8 (CCK-8; incubated with different concentrations of 25BM@HA). (C) Live staining of Cal-27 cells (scale bar = 100 μm). (D) CCK-8 assay of Cal-27 cells incubated with 25BM@HA under hypoxic conditions. (E) Colony formation assay of Cal-27 cells. (F and I) Fluorescence images (scale bar = 100 μm) and flow cytometry of the Cal-27 cells stained with DCFH-DA and tris(4,7-diphenyl-1,10-phenanthroline)ruthenium(II) dichloride ([Ru(dpp)_3_]Cl_2_). (G and H) The HIF-1α, Nrf2, and HO-1 protein expressions of Cal-27 cells were determined by western blot. *P < 0.05; **P < 0.01; ***P < 0.001. FITC, fluorescein isothiocyanate.

HA acts as a ligand for the CD44 protein, which is highly expressed on the surface of various tumor cells, including OSCC cells [30] and breast cancer cells [31]. HA-modified nanoparticles can target tumors overexpressing CD44 [32]. Therefore, in this study, HA was selected as a specific targeting moiety for tumor cells. The Cal-27, SAS, and SCC-9 OSCC cells, which express high levels of CD44 [30], were selected as the experimental models, and L929 cells, which express low levels of CD44 [33], served as the control, to explore the uptake of 25BM@HA by OSCC cells. After incubating 50 μg ml^−1^ 25BM@HA with these cells for 6 h, optical microscope observations revealed distinct black aggregates in Cal-27, SAS, and SCC-9 cells, indicating the uptake and accumulation of 25BM@HA, whereas almost no aggregation was observed in L929 cells (Fig. S6). Furthermore, 25BM@HA was labeled with FITC, and fluorescence detection was performed using flow cytometry. The results showed a significant increase in fluorescence in Cal-27, SAS, and SCC-9 cells, while there was no significant enhancement in fluorescence in L929 cells (Fig. 4A), confirming the active targeting capability of 25BM@HA toward OSCC cells.

After that, the antitumor activity of 25BM@HA was investigated. As illustrated in Fig. 4B and Fig. S7A, after stimulating tumor cells (Cal-27 and SAS) with 25BM@HA (0 to 50 μg ml^−1^) for 24 h without NIR irradiation, the viability of Cal-27 and SAS cells remained above 80%. However, after 5 min of NIR laser irradiation (at 37 °C), 40 μg ml^−1^ 25BM@HA significantly inhibited tumor cell growth, reducing the cell viability of Cal-27 and SAS to 46.4% and 50.3%, respectively. Moreover, the 50 μg ml^−1^ concentration further decreased cell viability to less than 20%. To eliminate the photothermal effect of 25BM@HA, analysis was conducted at a low temperature (4 °C). Under these conditions, 40 μg ml^−1^ 25BM@HA showed no significant inhibition of cell viability. However, 50 μg ml^−1^ reduced the viability of Cal-27 and SAS to 45.3% and 46.7%, respectively, which was less effective compared to the NIR laser irradiation group at 37 °C. The observed findings indicated that the antitumor effect of 25BM@HA was caused by the combination of PTT and PDT. Therefore, a concentration of 50 μg ml^−1^ was selected for subsequent experiments to further investigate the combined therapeutic effects of PTT and PDT. Both live-cell staining and colony formation assay confirmed that combined treatment with 25BM@HA and laser significantly inhibited tumor cell proliferation (Fig. 4C and E and Figs. S7B to D and S8).

Furthermore, the potential mechanisms through which 25BM@HA exerts its antitumor effects were investigated. The generation of ROS, critical for PDT treatment, was assessed in this study by using DCFH-DA to detect intracellular ROS production. Compared to the 25BM@HA group, the 25BM@HA + NIR group exhibited stronger green fluorescence (Fig. 4F and Fig. S7E). This results were consistent with those of flow cytometry (Fig. 4I and Fig. S7F). These findings indicate that NIR irradiation effectively stimulates the generation of intracellular ROS. After PDT, elevated ROS levels trigger oxidative stress, which induces cytotoxicity and DNA damage, ultimately resulting in cell death [34]. The Nrf2/HO-1 pathway plays an important role in regulating oxidative stress, with Nrf2 serving as a key regulator of the antioxidant response. Excessive ROS stimulation leads to an increase in Nrf2 expression [35]. Meanwhile, HO-1, a downstream target protein of Nrf2, can be up-regulated in response to stress conditions through Nrf2 activation [36]. In the current study, treating tumor cells with 25BM@HA + NIR resulted in a significant increase in the expression levels of Nrf2 and HO-1 (Fig. 4G and H and Fig. S7H and I), indicating that 25BM@HA induces cell death by stimulating oxidative stress response after exposure to 808-nm laser. Cheng et al. [37] prepared Bi_2_S_3_@Bi nanoparticles for the treatment of hypoxic tumors, finding that after 808-nm laser irradiation, the expression levels of Nrf2 and HO-1 proteins were significantly increased, consistent with the findings observed in the current study. Similarly, the expression of Nrf2 and HO-1 proteins significantly increased after radiotherapy [38]. However, the Nrf2/HO-1 pathway has a dual role: serving as a critical regulatory signal in the body’s antioxidant defense system while also being involved in the response to oxidative stress. Elevated levels of Nrf2 and HO-1 may enhance the antioxidant potential of tumor cells, shifting them from a pro-oxidative to an antioxidative state, thereby leading to the resistance of cancer cells to radiotherapy, chemotherapy, and even PDT [39,40]. A research study has shown that knocking down Nrf2 or HO-1 expression increases the radiosensitivity of cancer cells [41], further supporting this perspective.

The generation of ROS depends on the availability of O_2_ in the tumor microenvironment. Sufficient oxygen supply can alleviate tumor hypoxia and improve the effectiveness of PDT. The investigation focused on whether NIR irradiation induces 25BM@HA to decompose water within tumor cells to generate O_2_. For this purpose, [Ru(dpp)_3_]Cl_2_ was used to detect the O_2_ levels in cells as its red fluorescence can be quenched by O_2_. In the absence of 808-nm laser irradiation, the red fluorescence intensity of the 25BM@HA group was similar to that of the control group, indicating that the tumor cells were in a hypoxic state. After 808-nm laser irradiation, the fluorescence intensity of the NIR group was comparable to that of the control group, while the red fluorescence of the 25BM@HA + NIR group was significantly quenched (Fig. 4F and Fig. S7E). The flow cytometry results supported these findings (Fig. 4I and Fig. S7G), suggesting that 25BM@HA has a good oxygen supply capacity and can alleviate hypoxia. Furthermore, hypoxia, a hallmark of the tumor microenvironment in most solid tumors, can activate the expression of HIF-1α. Hypoxia influences tumor growth, metastasis, and treatment resistance by regulating pathways associated with cell metabolism, oxidative stress, angiogenesis, and immune evasion [42]. HIF-1α is highly expressed in various malignancies, such as breast cancer, esophageal cancer, and OSCC, and has emerged as a key target for cancer therapy [43]. Western blot results showed the expression of HIF-1α on both Cal-27 and SAS cells, with its expression significantly reduced after treatment with 25BM@HA + NIR (Fig. 4G and H and Fig. S7H and I), further indicating that 25BM@HA can supply oxygen and alleviate hypoxia. It also suggests that 25BM@HA may inhibit tumor growth through HIF-1α-related pathways. Interestingly, studies have indicated that down-regulating Nrf2 expression in tumor cells can inhibit HIF-1α accumulation under hypoxic conditions, thereby inhibiting HIF-1α-dependent tumor angiogenesis and metabolic metastasis [44]. Therefore, targeting Nrf2 in combination with phototherapy emerges as a highly promising therapeutic strategy. It not only suppresses antioxidant responses, promotes sustained ROS generation, and increases the sensitivity of hypoxic tumors to PDT but also disrupts the HIF-1α pathway, thereby synergistically enhancing the therapeutic effect.

Moreover, the antitumor effect of 25BM@HA combined with laser treatment was evaluated on tumor cells (Cal-27) under hypoxic conditions. The results showed that 25BM + NIR reduced the cell viability of Cal-27 to 37.4% (Fig. 4D) while simultaneously enhancing the generation of ROS and O_2_ (Fig. 4F and I). These results suggest that 25BM + NIR can overcome the limitations posed by hypoxia in PDT and demonstrate good antitumor effects.

### In vivo antitumor effect of 25BM@HA

**Fig. 5.**
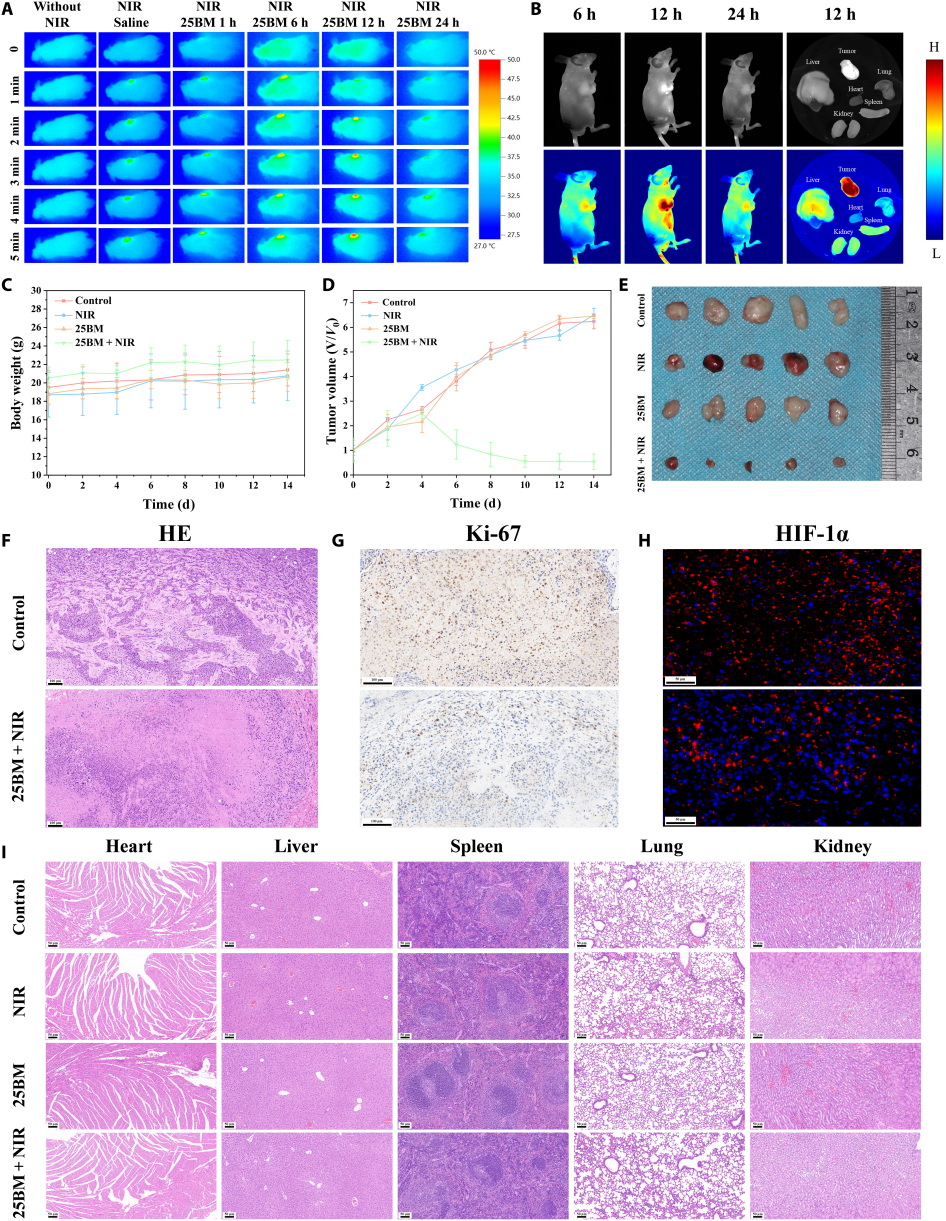
In vivo tumor accumulation and phototherapy of 25BM@HA in SAS-tumor-bearing mice. (A) In vivo thermal images of a tumor-bearing mouse after injection with saline and 25BM@HA under 808-nm laser irradiation (0.75 W cm^−2^, 5 min). (B) In vivo distribution after an injection with FITC-25BM@HA. (C) Body weight change of mice during 14 d of different treatments. (D) Relative tumor volume change of mice during 14 d of different treatments. (E) The photographs of tumors dissected at 14-d treatment. (F) Hematoxylin and eosin (HE) staining sections (scale bar = 100 μm), (G) immunohistochemistry (IHC) staining of Ki-67 (scale bar = 100 μm), and (H) immunofluorescence (IF) staining of the HIF-1α (scale bar = 50 μm) of tumor tissues after 14 d of treatment. (I) HE-stained images of the heart, liver, spleen, lung, and kidney from the different groups at 14-d treatment (scale bar = 50 μm).

To further evaluate the antitumor effect of 25BM@HA, a tumor model was developed in nude mice using SAS cells. The mice were randomly assigned to 4 groups: control group, NIR group, 25BM group, and 25BM + NIR group. Physiological saline and a saline solution of 25BM@HA were administered via tail vein injection every 2 d, with a dose of 10 mg kg^−1^ per injection. The phototherapy groups were irradiated with an 808-nm laser for 5 min at a power density of 0.75 W cm^−2^. Initially, the temperature change in the tumors of the nude mice was recorded using a photothermal imager after tail vein injection. The results indicated that after 5 min of irradiation with the 808-nm laser, the tumor temperature in the saline-injected group increased to less than 39 °C. However, after injecting 25BM@HA, the tumor temperature displayed a prolonged increase, reaching 48 °C at 12 h postinjection, before gradually decreasing by 24 h postinjection (Fig. 5A). This indicates that 25BM@HA can effectively target and accumulate in the tumor region following tail vein injection, with the highest accumulation occurring at 12 h, followed by slow metabolism from the body. The tumor-targeting and photothermal performance of 25BM@HA in animals confirms its potential for tumor phototherapy. Following the injection of FITC-modified 25BM@HA via the tail vein, in vivo imaging discovered the strongest fluorescence intensity in the tumor area at 12 h postinjection (Fig. 5B). Based on this finding, 808-nm laser irradiation was performed 12 h after the tail vein injection of 25BM@HA.

After 2 weeks of treatment, the tumor volumes in the control, NIR, and 25BM groups grew significantly, expanding to approximately 5 times their original size, while the tumor volume in the 25BM + NIR group decreased significantly (Fig. 5D). Corresponding images visually demonstrated the marked tumor suppression effect in the 25BM + NIR group (Fig. 5E). HE staining results revealed extensive damage and necrosis of tumor cells in the 25BM + NIR group (Fig. 5F). The expression level of Ki-67, a commonly used biomarker for cell proliferation, was evaluated. The IHC results showed that the expression level of Ki-67 in the 25BM + NIR group was significantly decreased (Fig. 5G and Fig. S9), indicating the improved antitumor performance of 25BM@HA. Immunofluorescence results further revealed that the expression of HIF-1α in the 25BM + NIR group was significantly lower compared to that in the control group (Fig. 5H and Fig. S10), consistent with the western blot in vitro results, indicating that 25BM@HA retained its oxygen-supplying capability in vivo, effectively alleviating tumor hypoxia following NIR laser irradiation.

Moreover, in vivo fluorescence imaging results showed enhanced fluorescence in the liver, spleen, and kidney regions following 25BM@HA treatment (Fig. 5B), suggesting that the compound was metabolized through these organs. HE staining analysis of the hearts, livers, spleens, lungs, and kidneys of mice belonging to different treatment groups showed no notable lesions (Fig. 5I). Furthermore, after 2 weeks of treatment, the body weight of the nude mice increased slightly (Fig. 5C), further supporting that 25BM@HA had favorable biosafety and was considered suitable for tumor phototherapy.

### Mechanism of 25BM@HA in tumor phototherapy

**Fig. 6.**
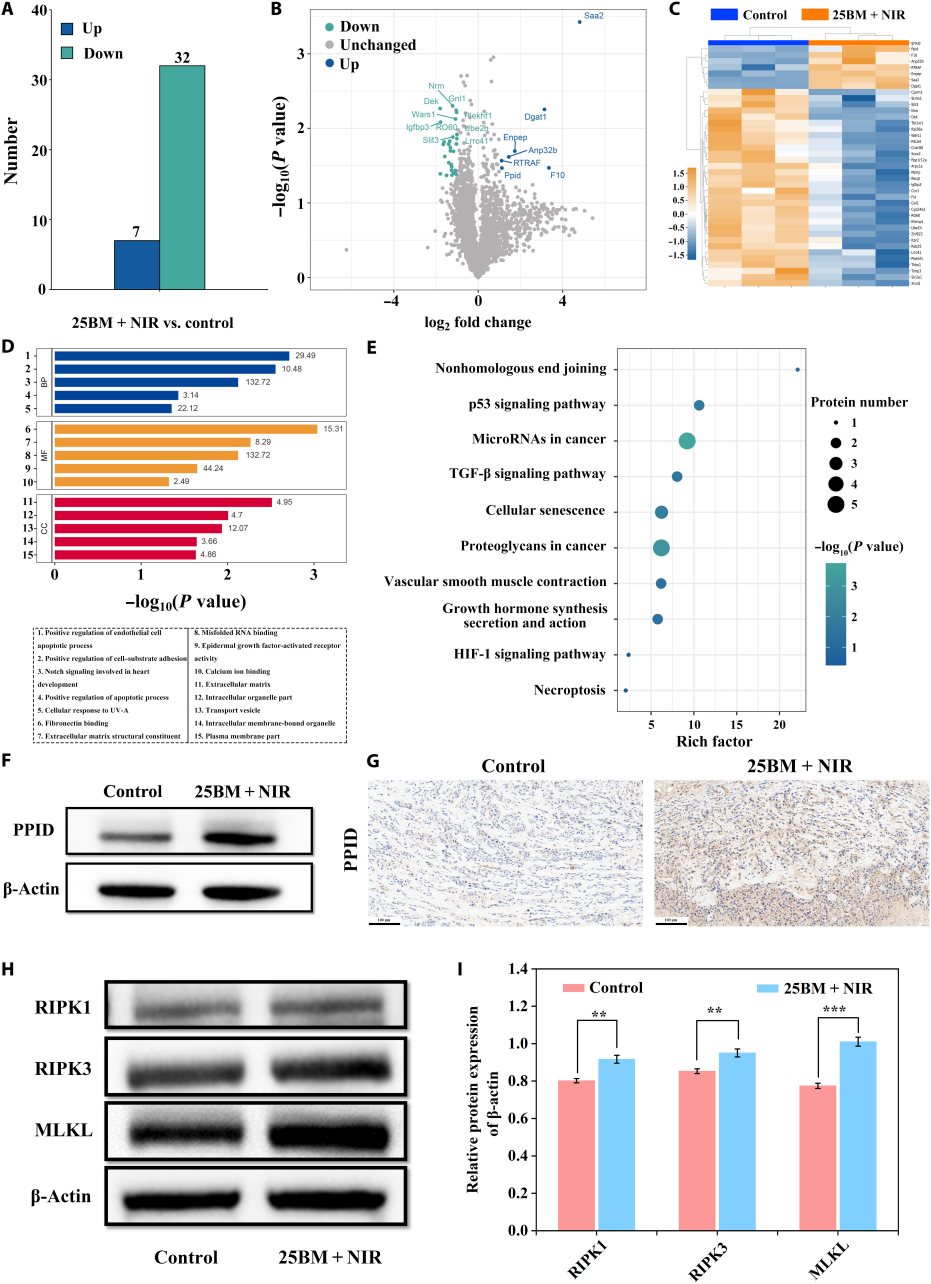
Mechanism exploration of 25BM@HA in tumor phototherapy by proteomic analysis. (A) The differentially expressed proteins in the control and 25BM + NIR groups. (B) The volcano map of identified differentially expressed proteins in the control and 25BM + NIR groups. (C) Heat maps displaying the differentially expressed proteins of different groups. (D) Gene Ontology (GO) pathway enrichment analysis. (E) Kyoto Encyclopedia of Genes and Genomes (KEGG) pathway enrichment analysis. (F) The PPID protein expression of SAS cells was determined by western blot. (G) The PPID protein expression of tumor tissues was detected by IHC (scale bar = 100 μm). (H and I) The RIPK1, RIPK1, and MLKL protein expressions of SAS cells were determined by western blot. *P < 0.05; **P < 0.01; ***P < 0.001. BP, Biological Process; MF, Molecular Function; CC, Cellular Component.

To further explore the potential mechanism of 25BM@HA in tumor phototherapy, proteomic analysis was carried out on the tumors of nude mice after treatment. As shown in Fig. 6A, the 25BM + NIR group displayed 39 differential proteins compared to the control group, with 7 up-regulated and 32 down-regulated proteins. A volcano plot and a heat map were constructed based on the differentially expressed proteins (Fig. 6B and C). Following this, Fisher’s exact test was applied for the GO and KEGG pathway enrichment analysis of the differential proteins. As shown in Fig. 6D and E, the 25BM@HA + NIR treatment was primarily enriched in pathways associated with the cell cycle and apoptosis/necrosis. The pathways include extracellular matrix, intracellular organelles, misfolded RNA binding, positive regulation of apoptosis, cellular response to UV-A, p53 signaling pathway, TGF-β signaling pathway, and cellular senescence. These findings align with previous transcriptomic analyses following PTT [45]. Particularly, in the GO enrichment analysis for “response to UV-A”, only one differential protein, PPID, was identified. In the KEGG pathway enrichment analysis, PPID was found to be enriched in pathways associated with cellular senescence and necroptosis. Therefore, 25BM@HA + NIR may inhibit tumor growth by up-regulating PPID expression, thereby inducing tumor cell senescence and necroptosis.

PPID, also known as cyclophilin 40 or cyclophilin D, is a cyclophilin protein located in the mitochondrial matrix. It serves as a gatekeeper for mitochondria, regulating their function and integrity. PPID is a critical regulator of cell death, while its increased phosphorylation can trigger the opening of the mitochondrial permeability transition pore (mPTP), thereby promoting necroptosis [46]. Studies have shown that compounds like bishonokiol A and 1,2-diarachidonoyl-*sn*-glycero-3-phosphoethanolamine can up-regulate PPID expression by activating the RIP1/RIP3/MLKL pathway, thereby promoting ROS production while facilitating mPTP-mediated tumor cell necrosis [47]. The activation of the PPID–mPTP axis represents a key mechanism through which various antitumor drugs induce tumor cell death [46]. Similarly, bromocriptine can promote PPID phosphorylation via the RIP3/MLKL pathway, promoting necrosis in prolactinomas [48]. Western blot of SAS cells (Fig. 6F and Fig. S11A) and IHC of the tumor model (Fig. 6G and Fig. S11B) showed that, compared to the control group, the 25BM + NIR group had a significantly higher level of PPID protein expression. As shown in Fig. 6H and I, the expression levels of RIPK1, RIPK3, and MLKL proteins in SAS cells were significantly increased after treatment with 25BM@HA combined with NIR laser, suggesting that 25BM + NIR can promote necroptosis. Therefore, it is hypothesized that 25BM@HA + NIR may trigger tumor cell necroptosis by activating the RIP1/RIP3/MLKL pathway, leading to increased PPID expression. Further investigations will be conducted to verify this pathway.

Interestingly, studies have demonstrated that inhibiting PPID expression significantly weakens the toxicity of chemotherapy drugs on tumors [49], while the overexpression of PPID significantly enhances cancer cell sensitivity to chemotherapy drugs and cytotoxicity [50]. Therefore, it can be inferred that overexpression of PPID may reduce tumor cell resistance to phototherapy, and combining treatments could better enhance the phototherapeutic effects of nanomaterials.

## Conclusion

In this study, we designed and synthesized the BM@HA semiconductor photosensitizer, which, under NIR laser irradiation, can produce O_2_, relieve tumor hypoxia, and enhance PDT efficacy. Combined with PTT, it achieved efficient treatment of OSCC. The formation of heterojunctions and the high photothermal conversion ability of BM@HA facilitated the effective separation of electron–hole pairs induced by NIR (808-nm laser). Furthermore, it also maintained the high reduction and oxidation ability of the separated charge carriers, promoting the generation of O_2_ and ROS. Surface modification of HA improved the biocompatibility and tumor-targeting efficiency, resulting in substantial tumor ablation when PDT and PTT were combined under 808-nm laser irradiation. Proteomic analysis identified PPID as a key differential protein, suggesting that 25BM@HA + NIR treatment may induce tumor cell necroptosis by activating PPID-related pathways. Inducing the expression of the key protein PPID in combination with phototherapy appears to be a more effective therapeutic strategy. In conclusion, the BM@HA semiconductor photosensitizer not only overcame the challenges posed by tumor hypoxia in PDT but also displayed improved anticancer activity when combined with PTT, highlighting its considerable potential for the treatment of OSCC.

## Data Availability

The full data used to support the findings of this study are available from the corresponding author upon request. All data generated or analyzed during this study are included in this published article.
