# Supplementary material for: Hyaluronic Acid-Functionalized Bismuth Vanadate/Molybdenum Disulfide Nanoheterojunctions Achieve Efficient Phototherapy of Hypoxic Tumor
Source: Biomater Res. 2025 Jul 18;29:0228. doi: 10.34133/bmr.0228 (PMC12271743; doi:10.34133/bmr.0228)

**Hyaluronic Acid-Functionalized Bismuth Vanadate/Molybdenum Disulfide Nanoheterojunctions Achieve Efficient Phototherapy of Hypoxic Tumor**

Yunqing Pang^1,2,3^, Jia Guo^2,3^, Qianlong Ma^2,3^, Jing Qi^1,2,3^, Lv Liu^2,3^, Yinzhong Bu^1,2,3^, Jing Wang^1,2,3*^

^1^ The First School of Clinical Medicine, Lanzhou University, Lanzhou, Gansu, 730000, China

^2^ School/Hospital of Stomatology, Lanzhou University, Lanzhou, Gansu, 730000, China

^3^ Clinical Research Center for Oral Diseases, Lanzhou, Gansu, 730000, China

Address correspondence to: J.W. (E-mail: [wangjing@lzu.edu.cn](mailto:wangjing@lzu.edu.cn))

**E-mail address**

Yunqing Pang [pangyunqing@126.com](mailto:pangyunqing@126.com)

Jia Guo [276141823@qq.com](mailto:276141823@qq.com)

Qian long Ma [maql18@lzu.edu.cn](mailto:maql18@lzu.edu.cn)

Jing Qi [qijing2003@foxmail.com](mailto:qijing2003@foxmail.com)

Lv Liu [bdkqll@163.com](mailto:bdkqll@163.com)

Yinzhong Bu [buyinzhong@163.com](mailto:buyinzhong@163.com)

Jing Wang [wangjing@lzu.edu.cn](mailto:wangjing@lzu.edu.cn)


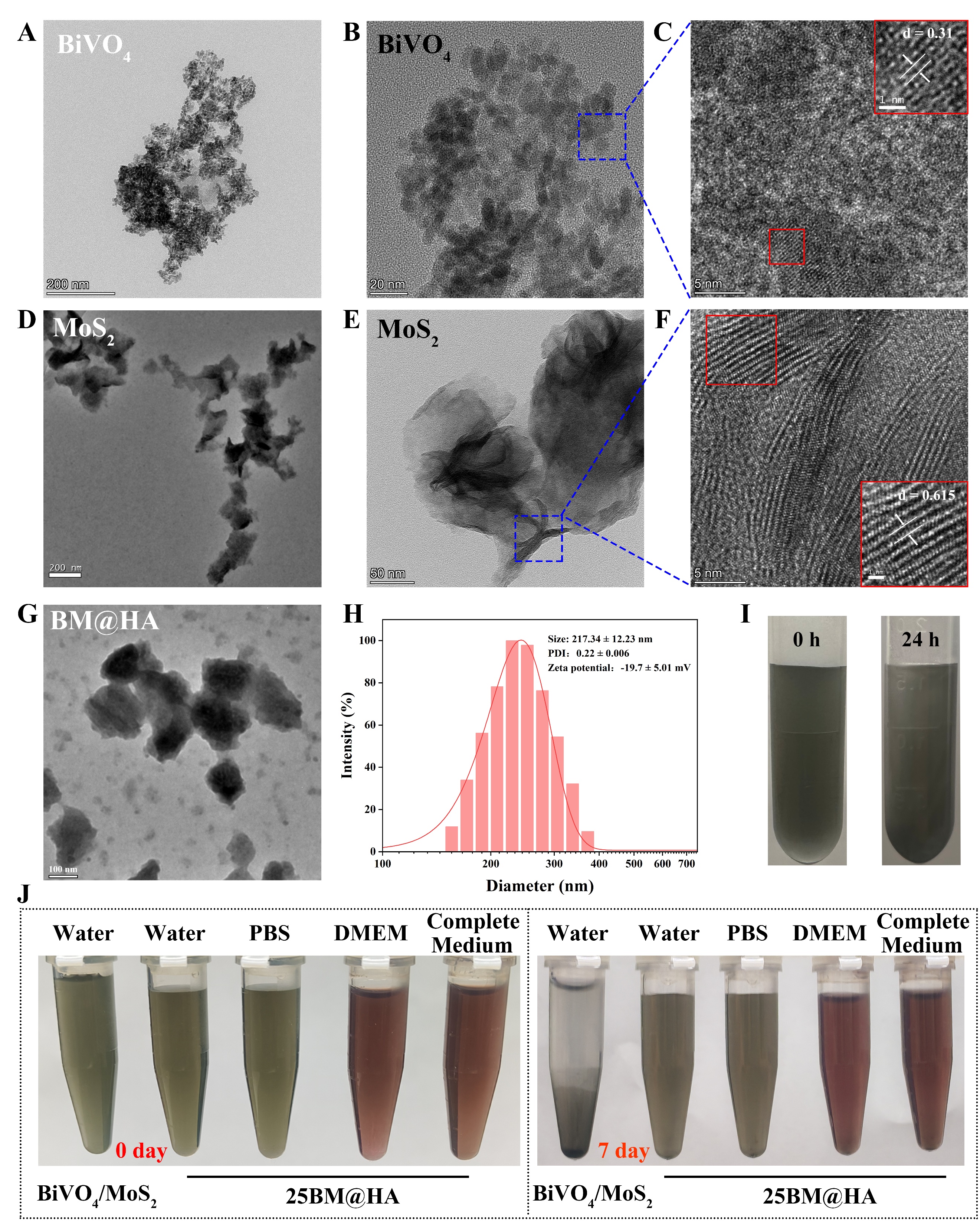


Figure S1. (A, B and C) TEM and HRTEM images of BiVO_4_. (D, E and F) TEM and HRTEM images of MoS_2_. (G) TME image of BM@HA. (H) The hydrodynamic diameter and the zeta potential of 25BM@HA dispersion in water. (I) Photo pictures of 25BM@HA dispersed in water after 24 h. (J) Photo pictures of BiVO_4_/MoS_2_ dispersed in water after 7 day and photo pictures of 25BM@HA dispersed in water, PBS, DMEM, complete medium after 7 day.


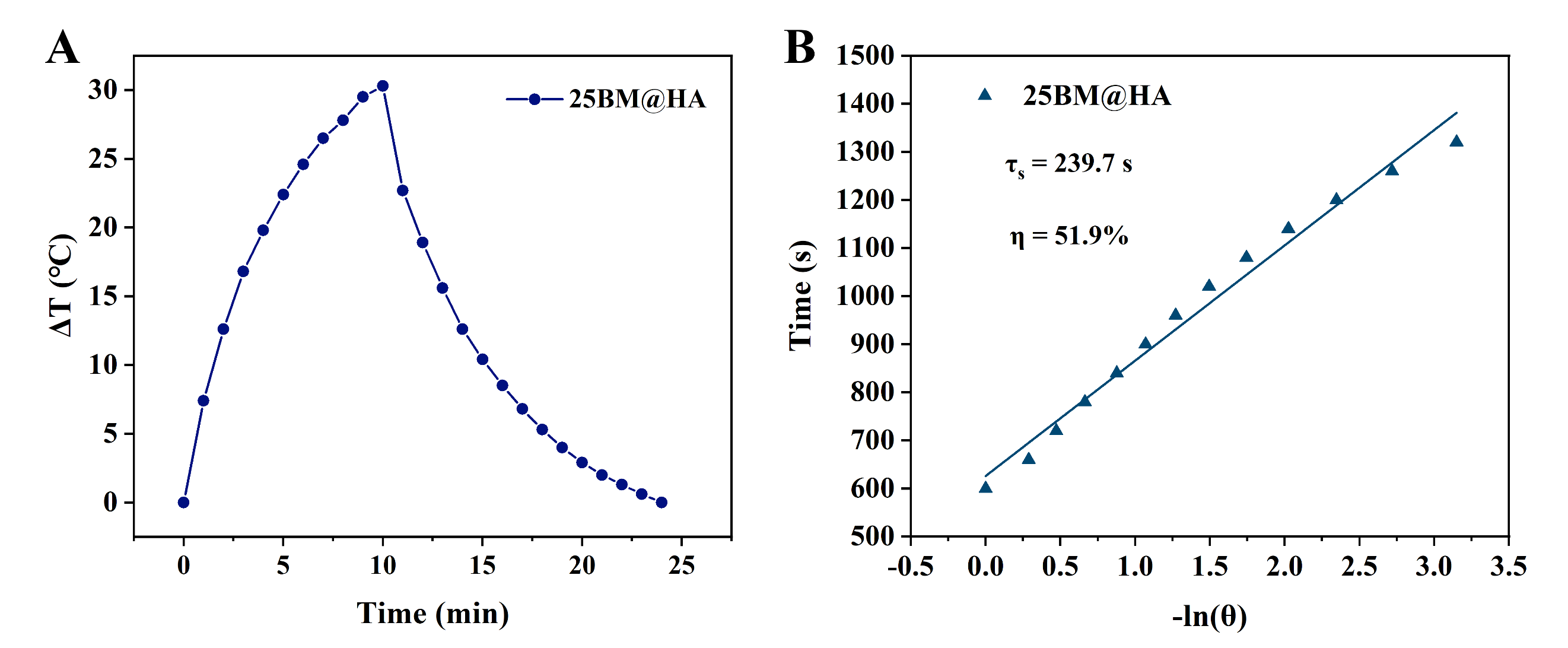


Figure S2. (A) Heating and cooling profiles of 25BM@HA (100 µg/mL) under 808 nm laser irradiation (0.75 W/cm^2^, 10 min). (B) Plot and linear fit of time versus negative natural logarithm of the temperature increment for the cooling rate of 25BM@HA aqueous suspensions.


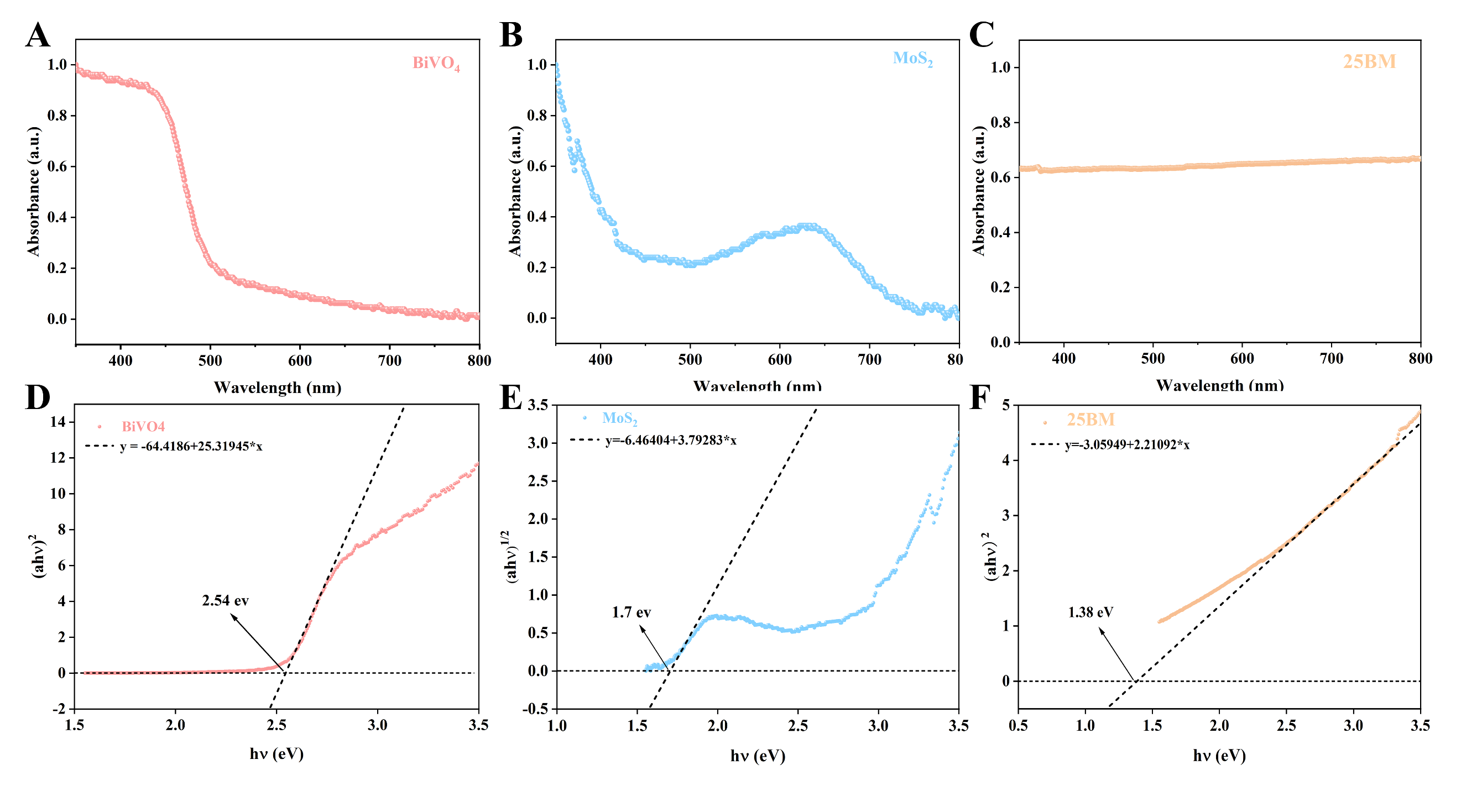


Figure S3. The UV-visible diffuse reflection spectroscopy of BiVO_4_ (A), MoS_2_ (B) and 25BM (C). The (αhν)^1/n^-hν curve of BiVO_4_ (D), MoS_2_ (E) and 25BM (F).


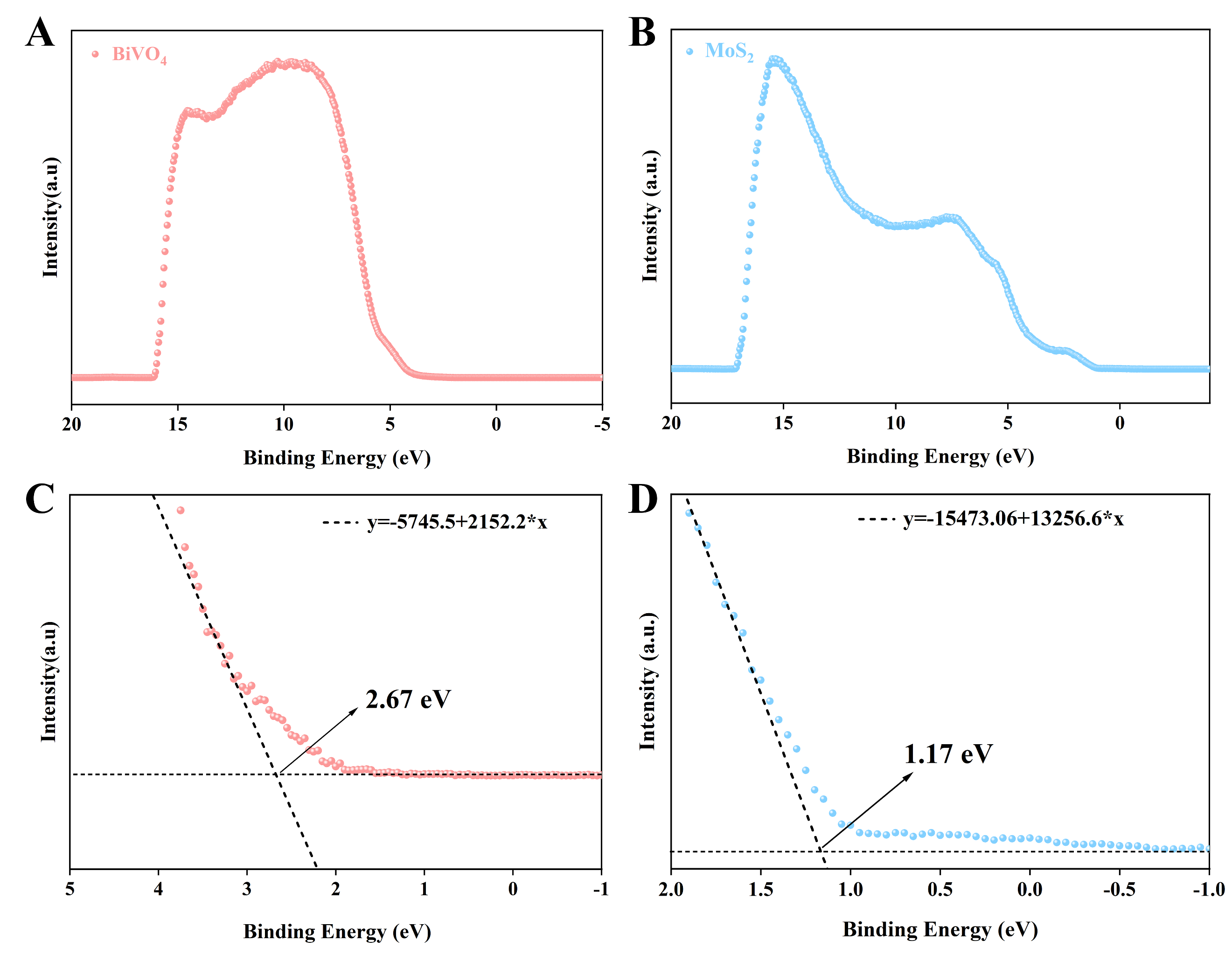


Figure S4. UPS spectrum of BiVO_4_ (A) and MoS_2_ (B). The valence band of BiVO_4_ (C) and MoS_2_ (D) were evaluated by UPS.


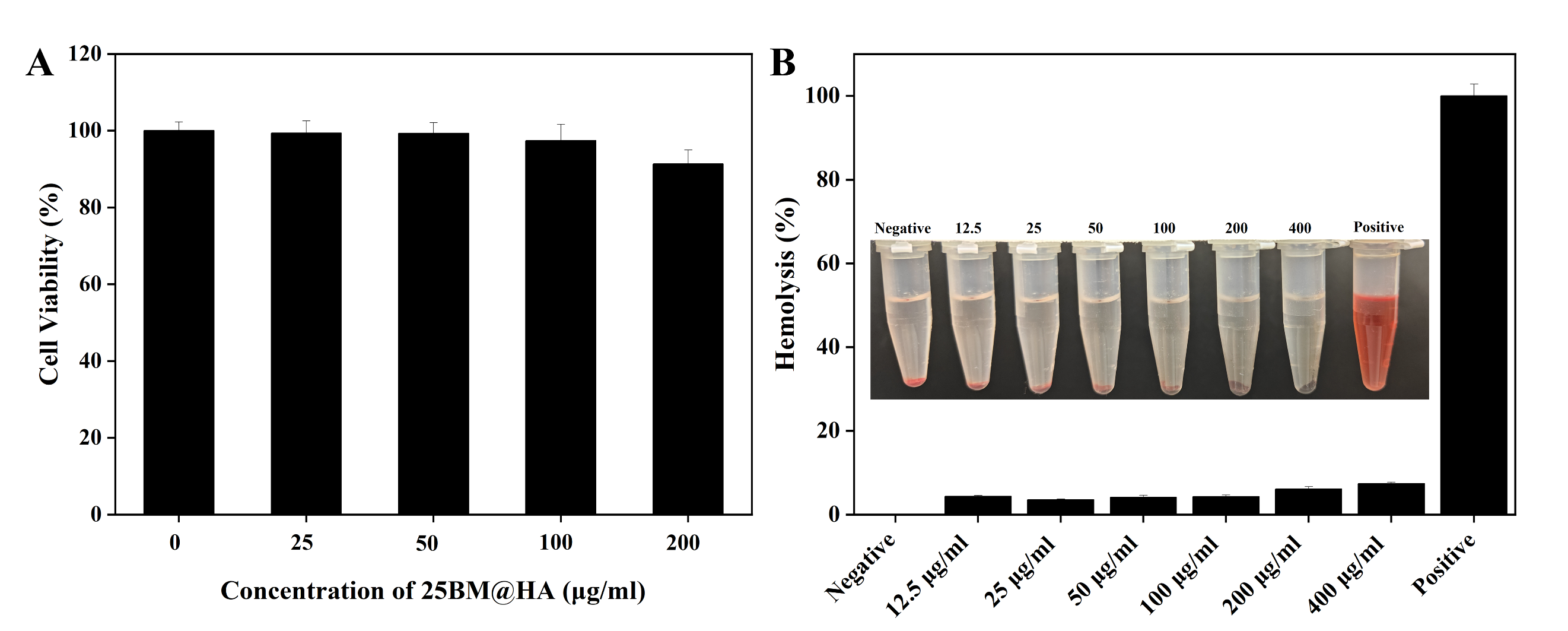


Figure S5. (A) Viability of L929 cells assessed by CCK-8, which incubated with different concentrations of 25BM@HA (0 - 200 μg/mL). (B) Hemolysis activities of 25BM@HA at different doses (0 - 400 μg/mL).


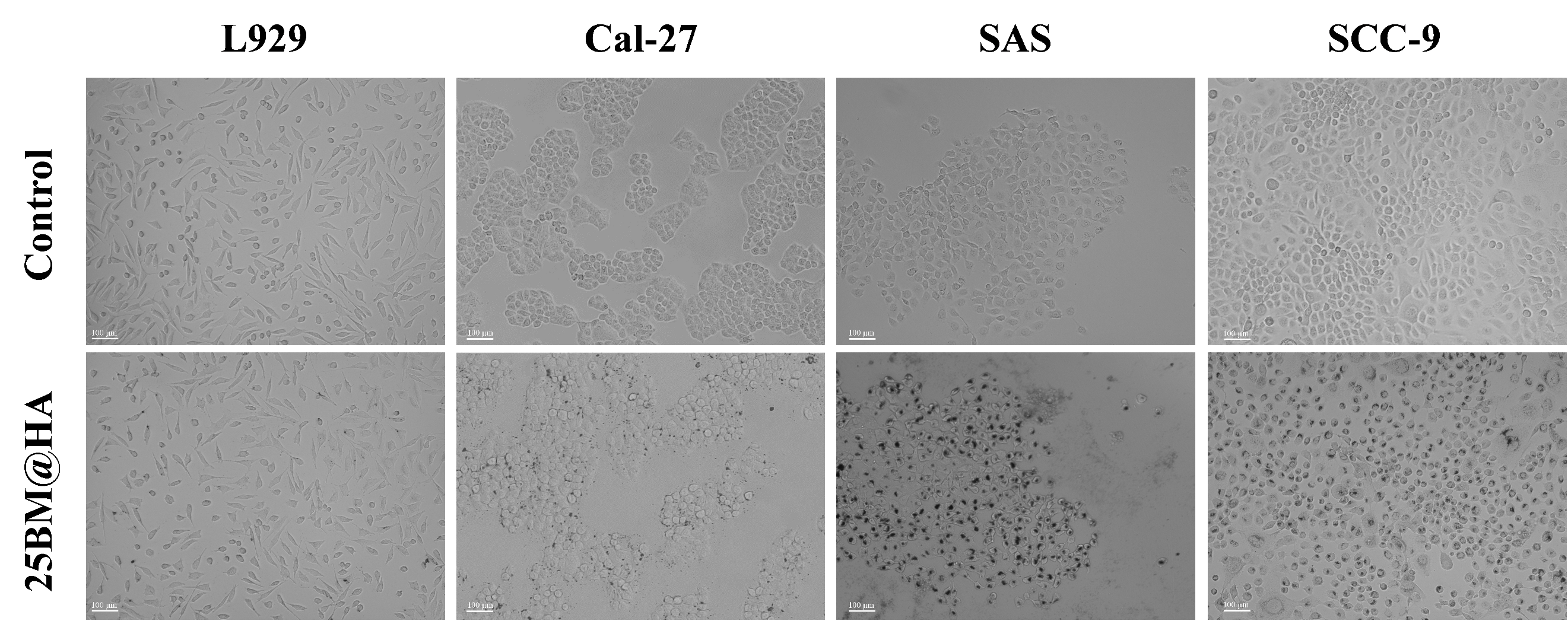


Figure S6. The microscopic images of cellular uptake behavior of 25BM@HA after incubation for 6 h in different cells (L929, Cal-27, SAS and SCC-9), scale bar = 100 μm.


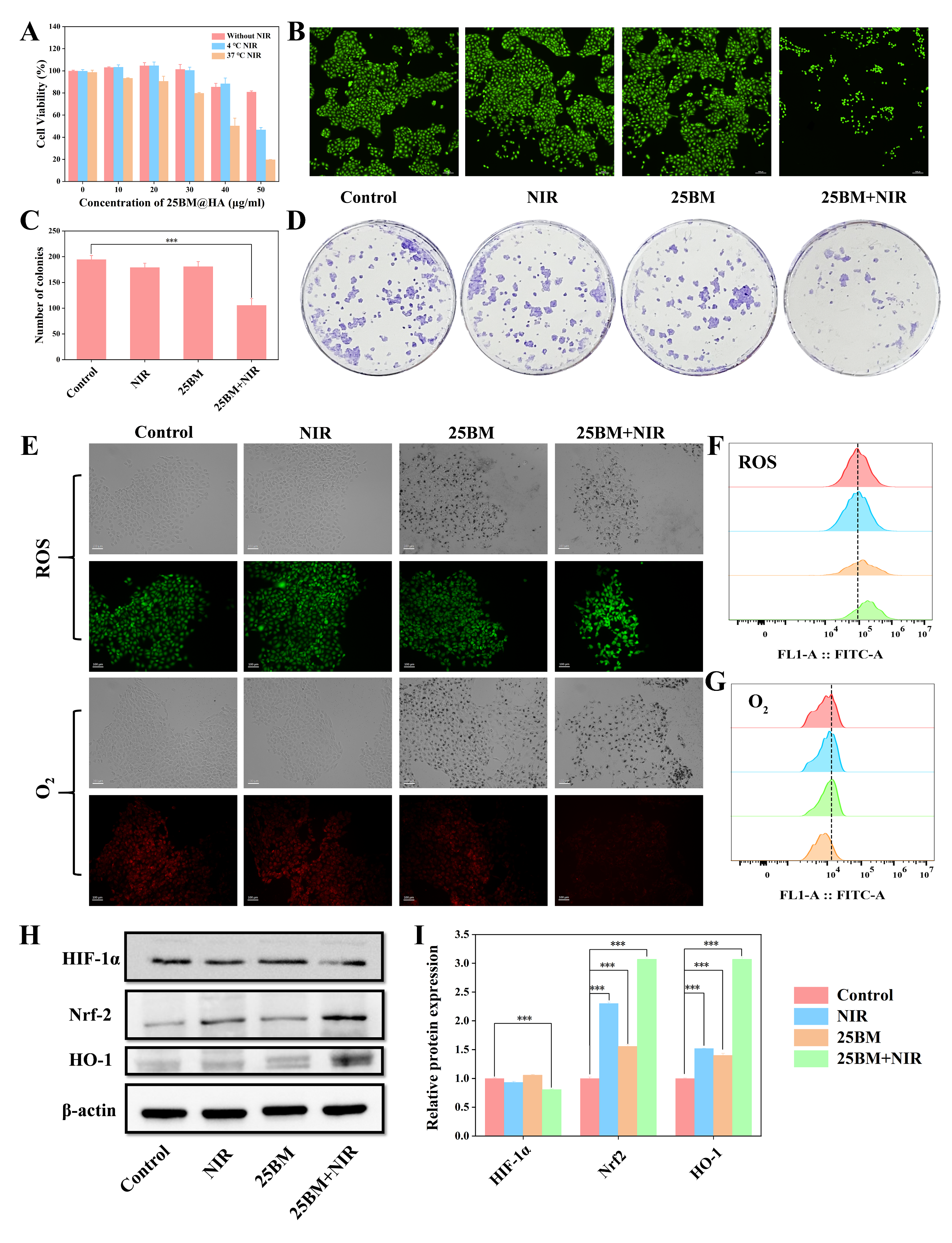


Figure S7. The effect of 25BM@HA on SAS cells. (A) Viability of SAS cells assessed by CCK-8 (incubated with different concentrations of 25BM@HA). (B) Live staining of SAS cells (scale bar = 100 μm). (C and D) Colony formation assay of SAS cells. (E, F and G) The microscopic images, fluorescence images and flow cytometry of SAS cells stained with DCFH-DA and [Ru(dpp)_3_]Cl_2_ (scale bar = 50 μm). (H and I) HIF-1α, Nrf2, and HO-1 protein expressions of SAS cells were determined by western blot.


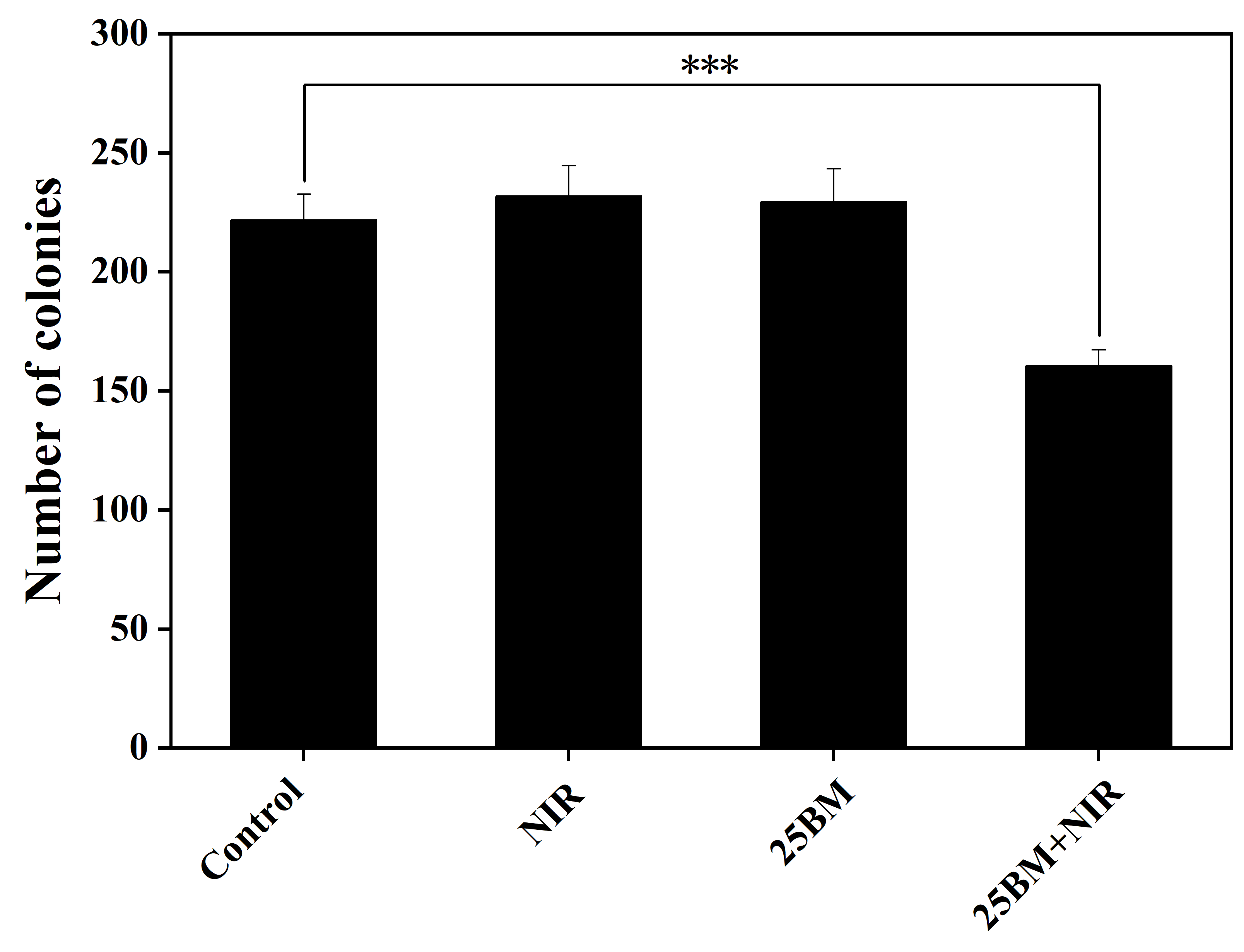


Figure S8. Quantitative analysis of clonal formation assays in SAS cells with different treatments. Data were presented as mean ± SD. **P<0.05, **P<0.01, ***P<0.001.*


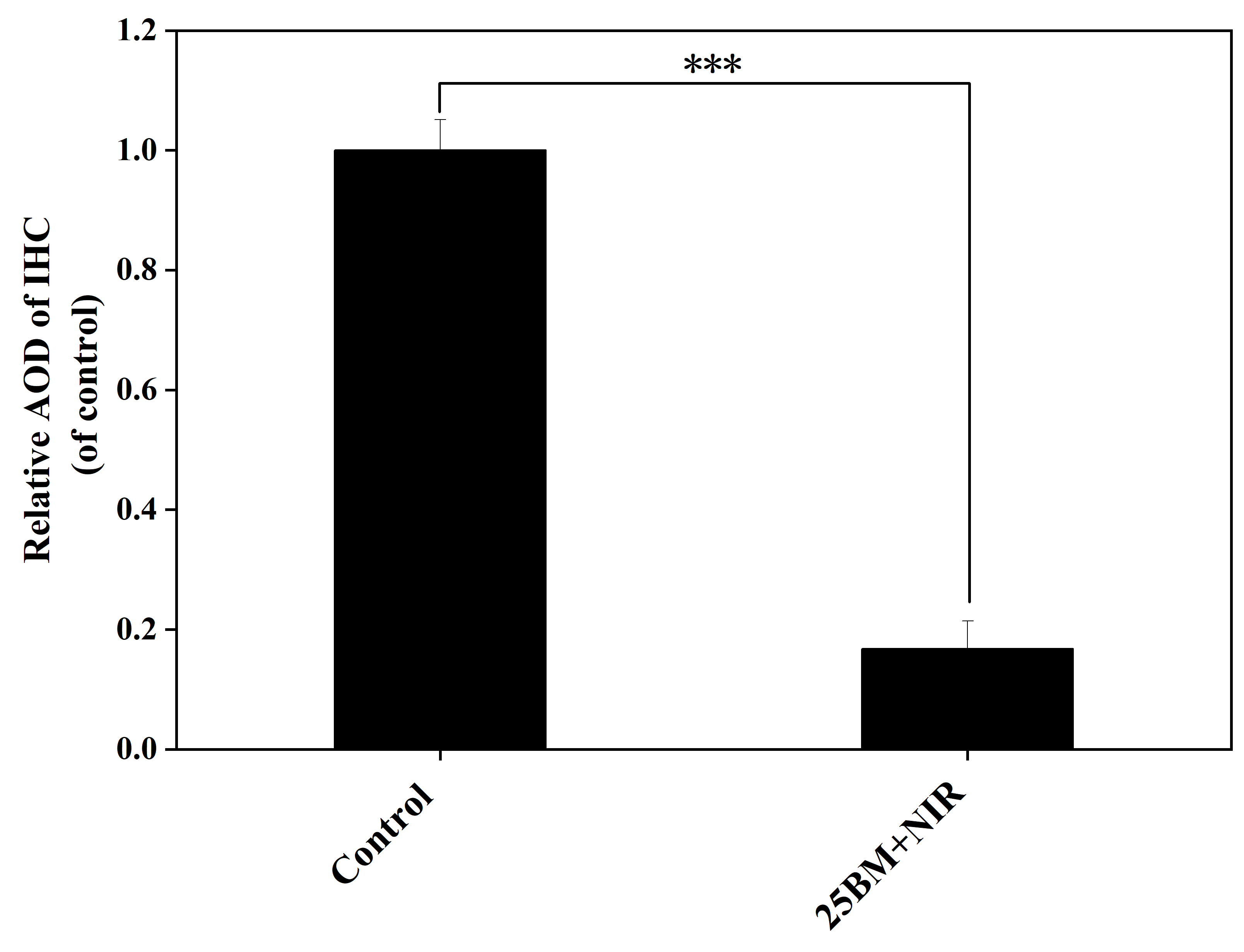


Figure S9. Quantitative analysis of the expression of Ki67 in tumor tissues were detected by IHC. Data were presented as mean ± SD. **P<0.05, **P<0.01, ***P<0.001.*


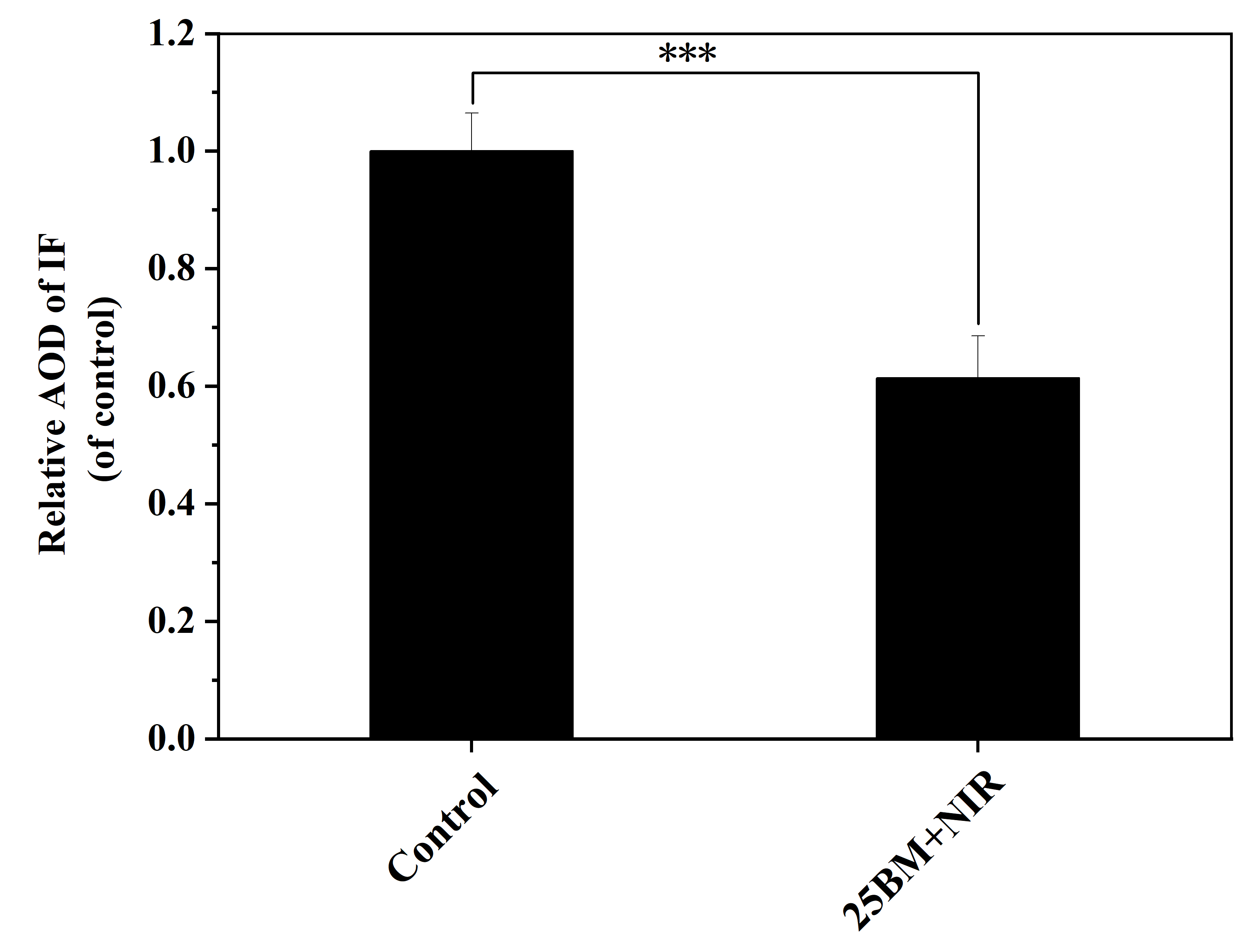


Figure S10. Quantitative analysis of the expression of HIF-1α in tumor tissues were detected by IF. Data were presented as mean ± SD. **P<0.05, **P<0.01, ***P<0.001.*


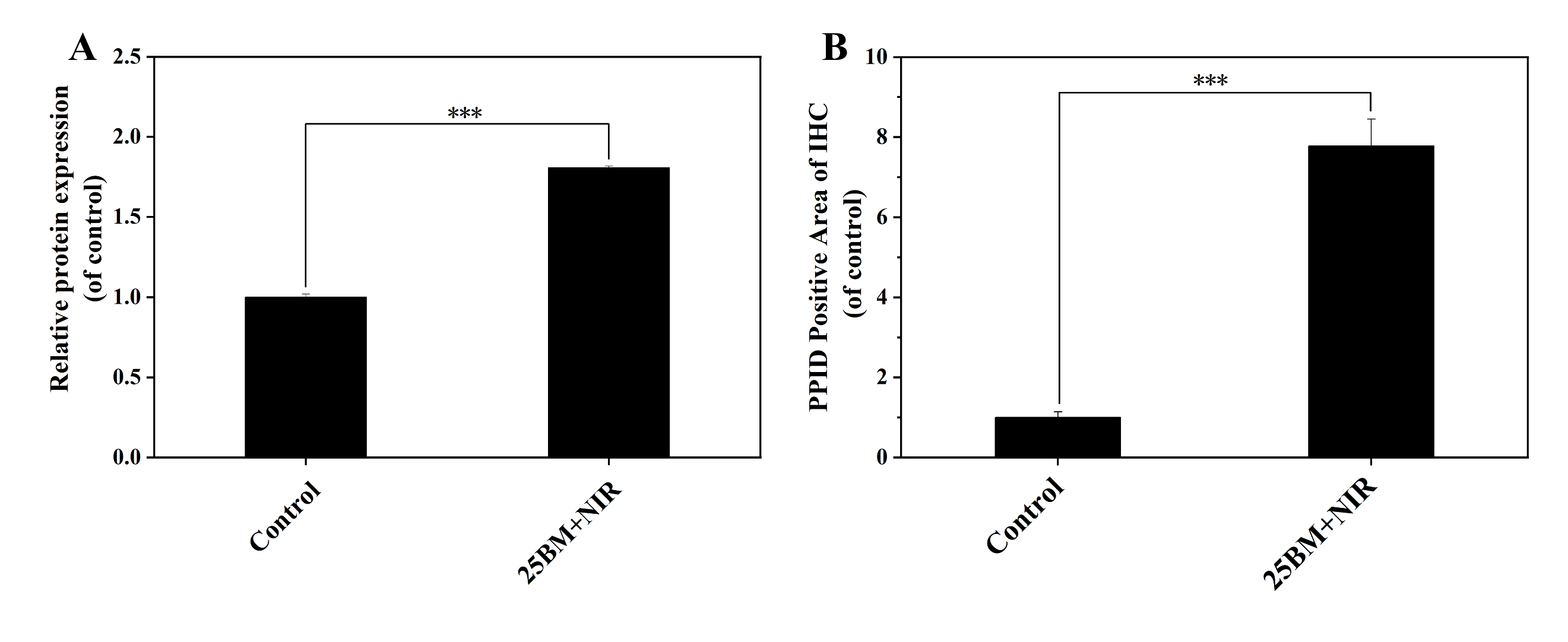


Figure S11. (A) Quantitative analysis of the expression of PPID in SAS cells were examined by western blot. (B) Quantitative analysis of the expression of PPID in tumor tissue (SAS) were examined by IHC. The data shown represent the mean ± SD. **P<0.05, **P<0.01, ***P<0.001.*

Table S1 Global New Cases and Deaths of oral cancer

| Year | NEW CASES | NO. OF  NEW CASES | DEATHS | NO. OF  DEATHS |
| --- | --- | --- | --- | --- |
| 2018 | 354,864 | 18 | 177,384 | 16 |
| 2020 | 377,713 | 18 | 177,757 | 17 |
| 2022 | 389,485 | 16 | 188,230 | 15 |

**Original images for gels and blots**

**Figure 4G**


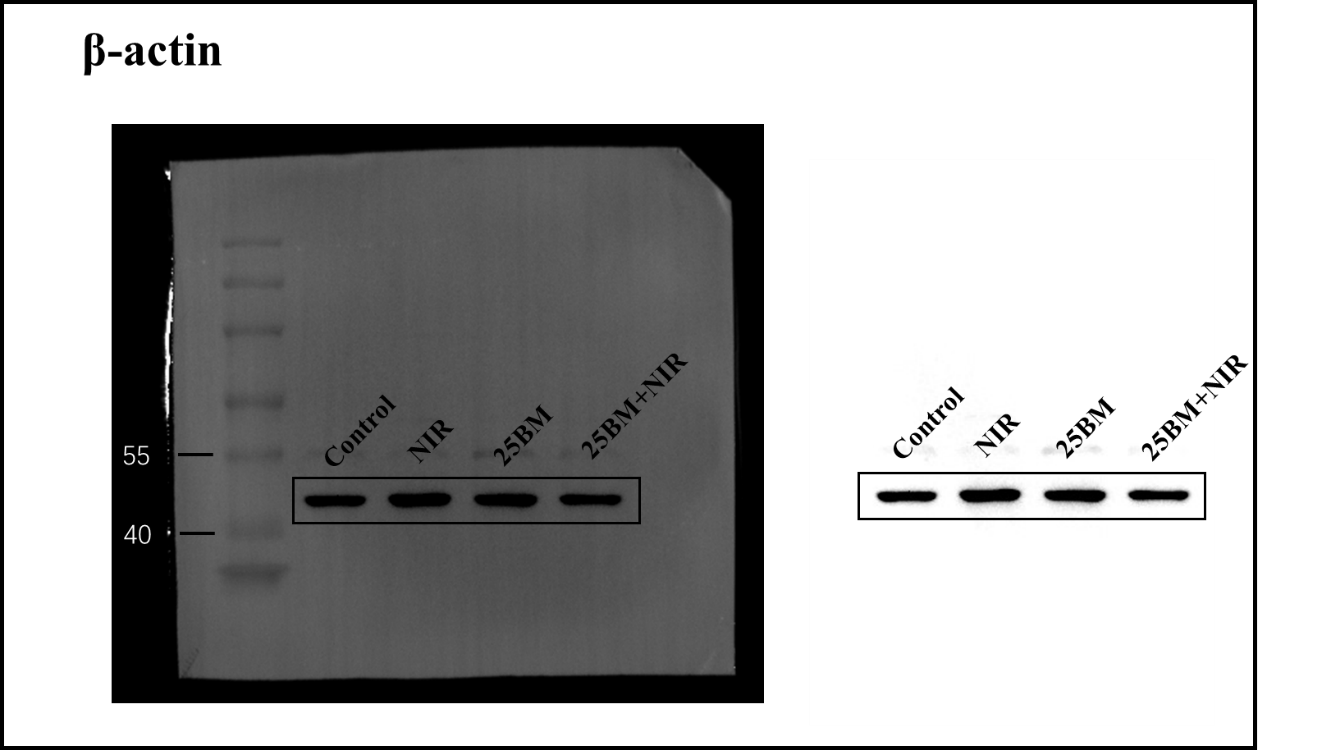


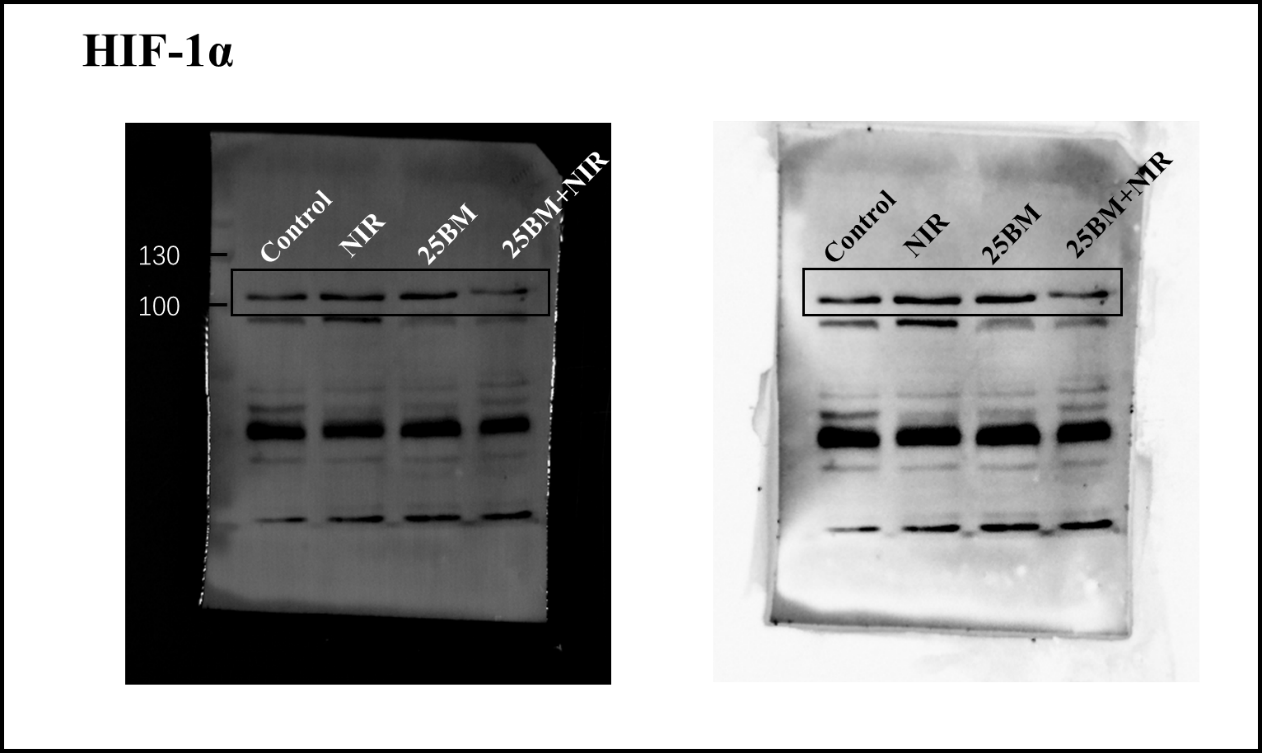


**Figure 4G**


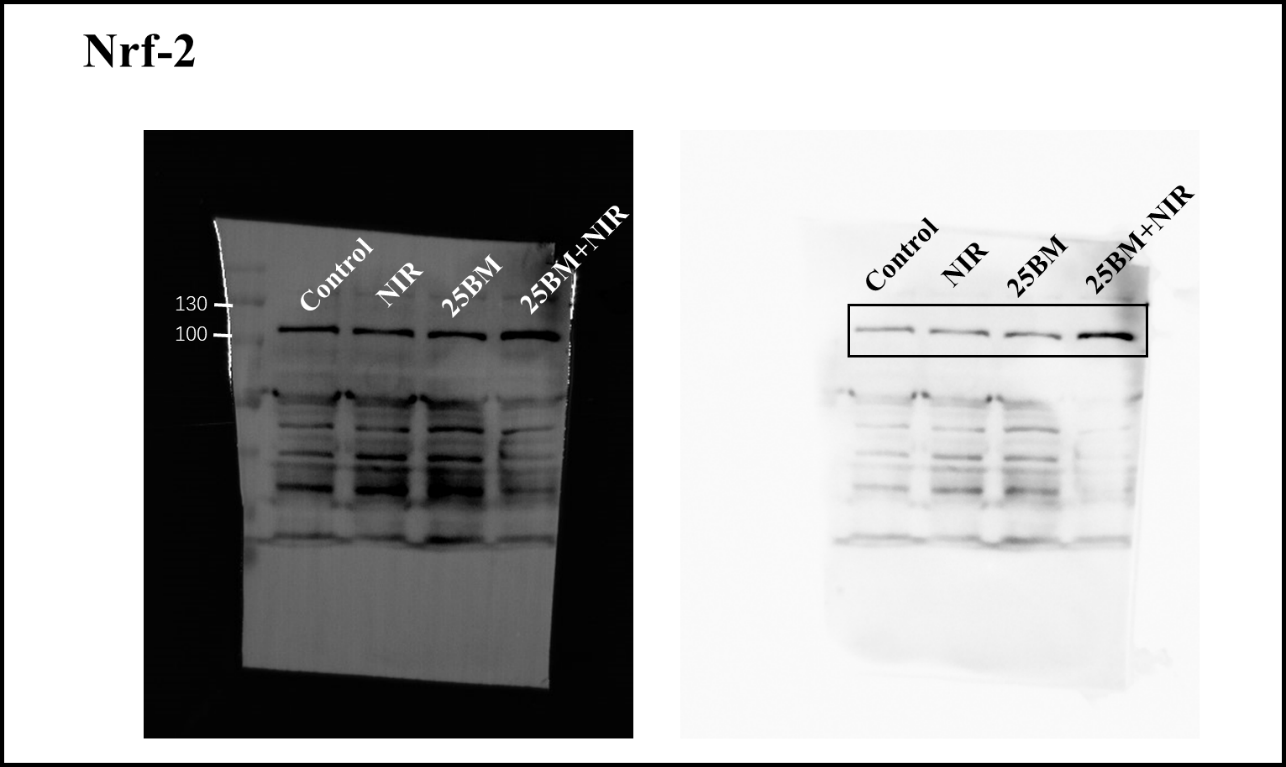


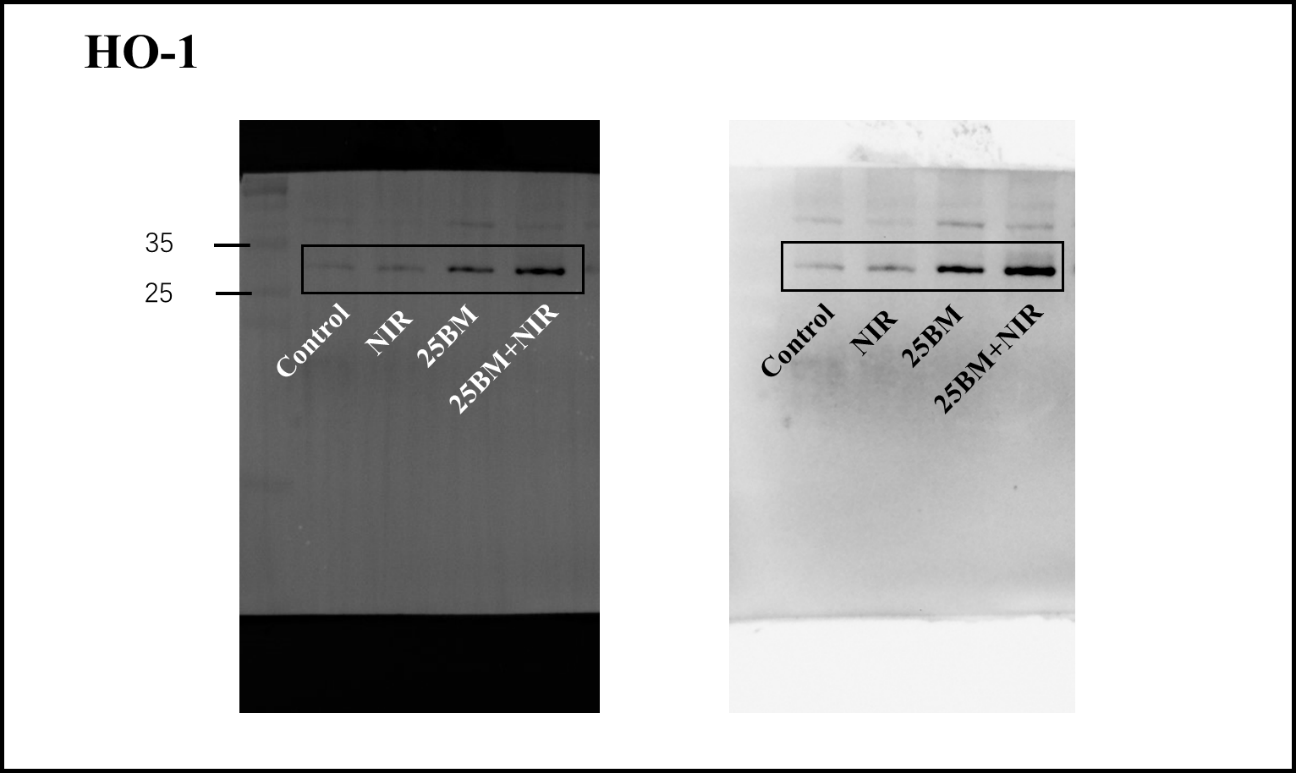


**Figure S8H**


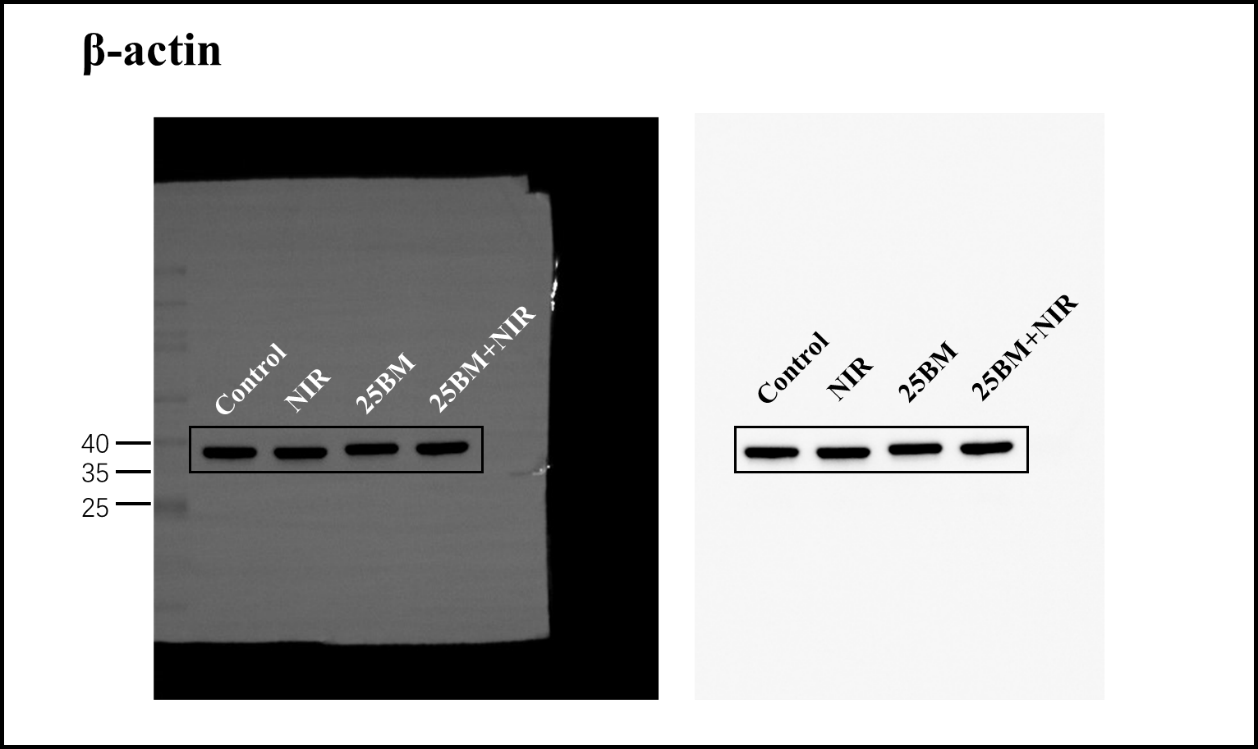


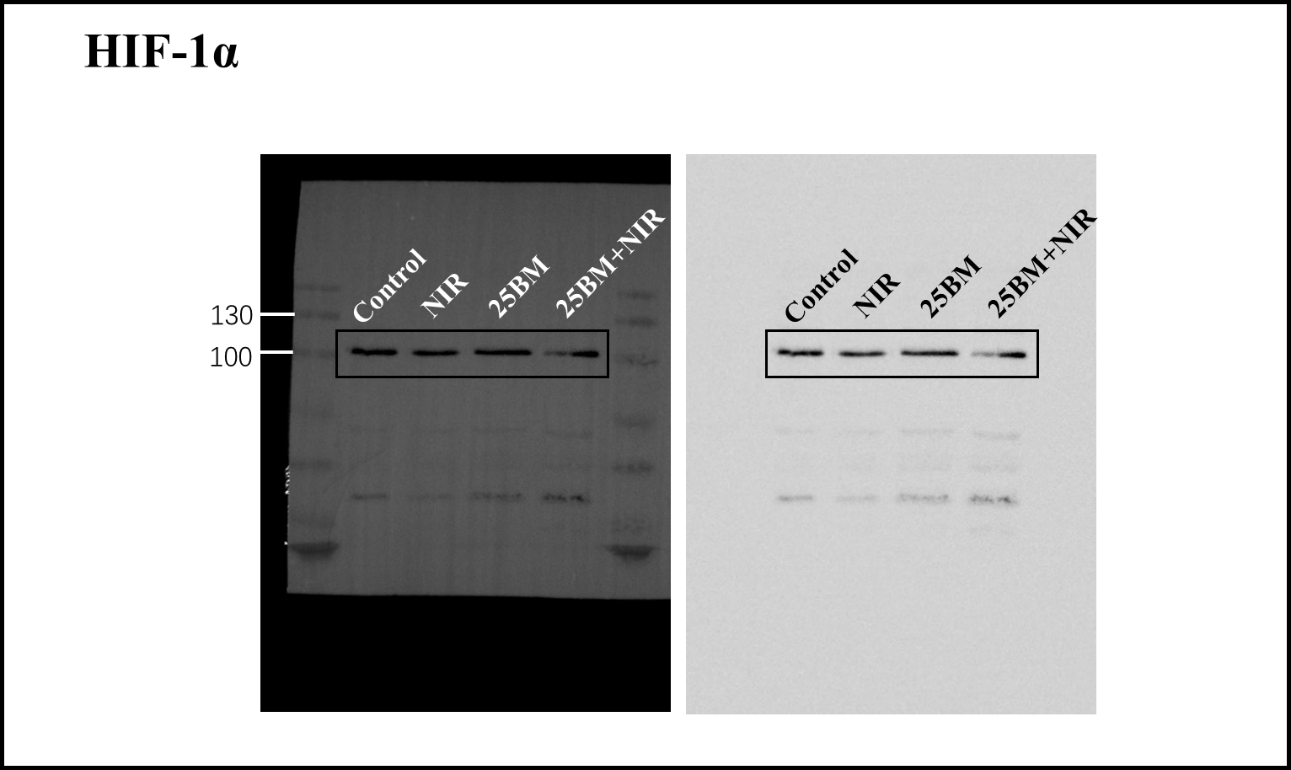


**Figure S8H**


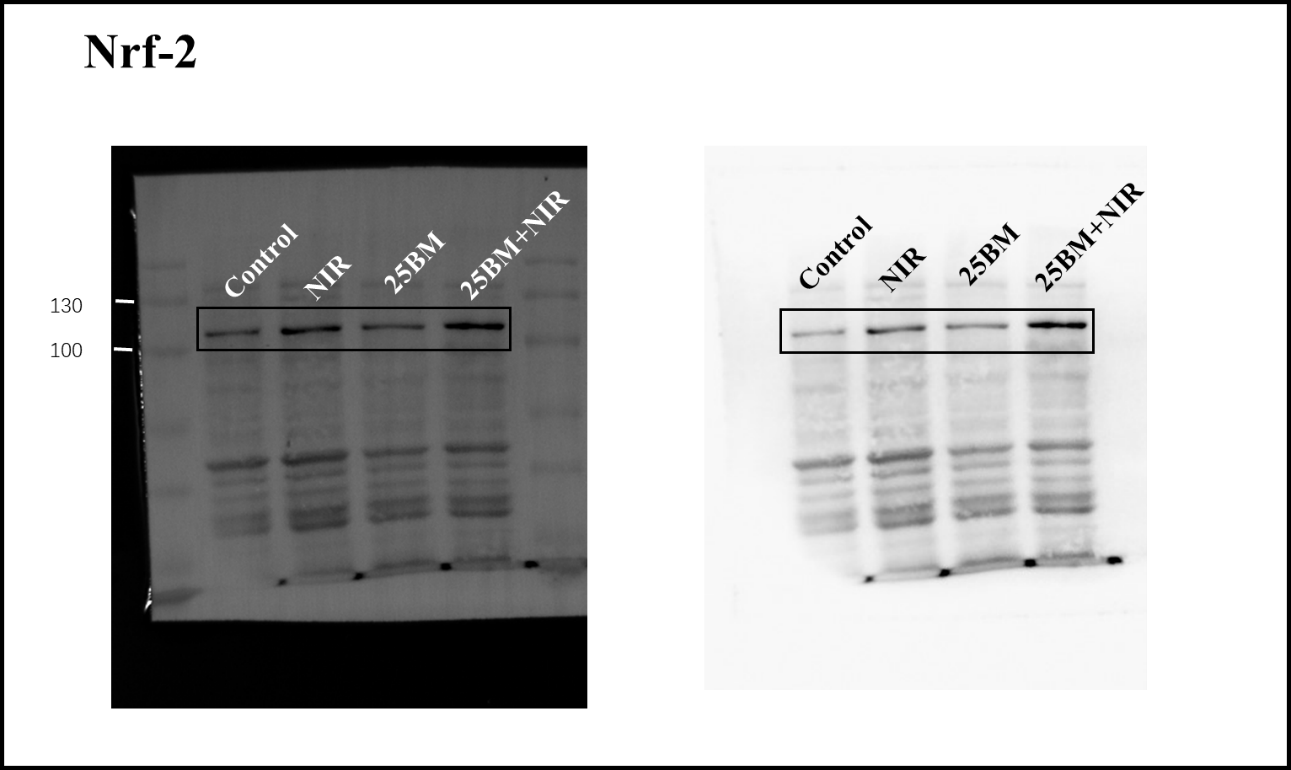


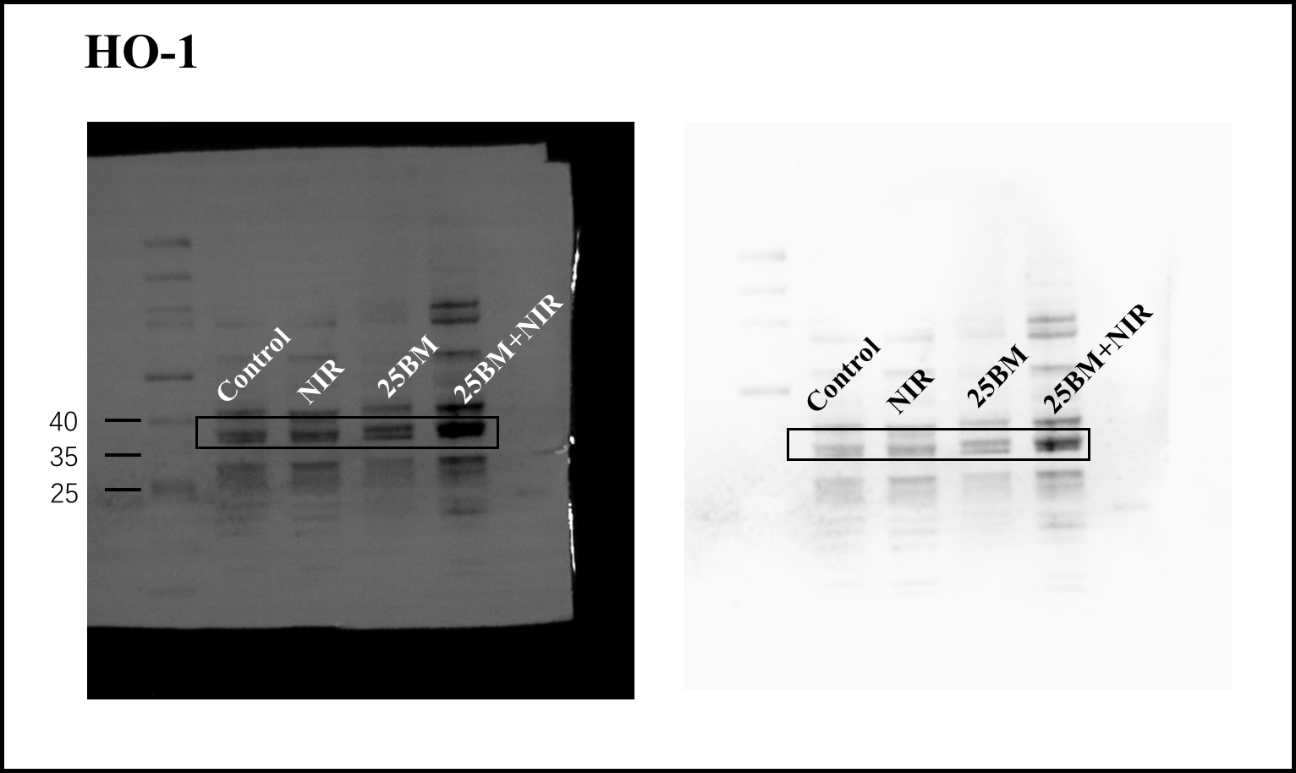


**Figure 5F**


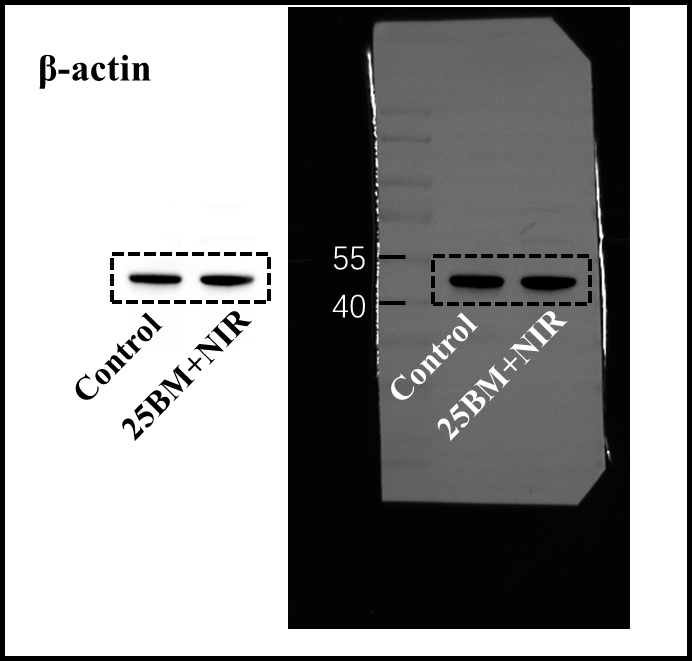


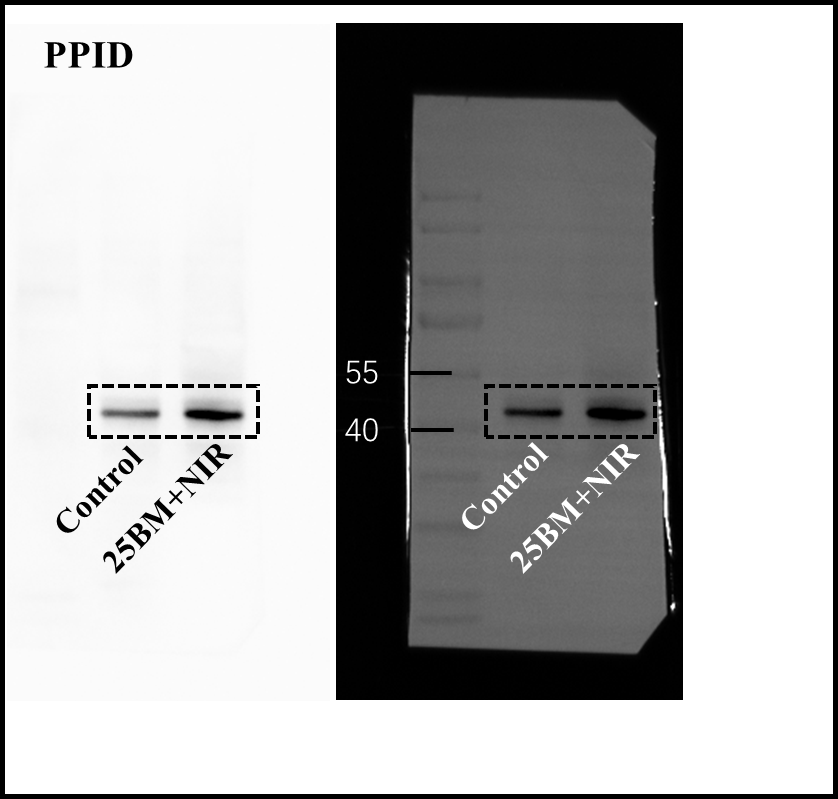


**Figure 5H**


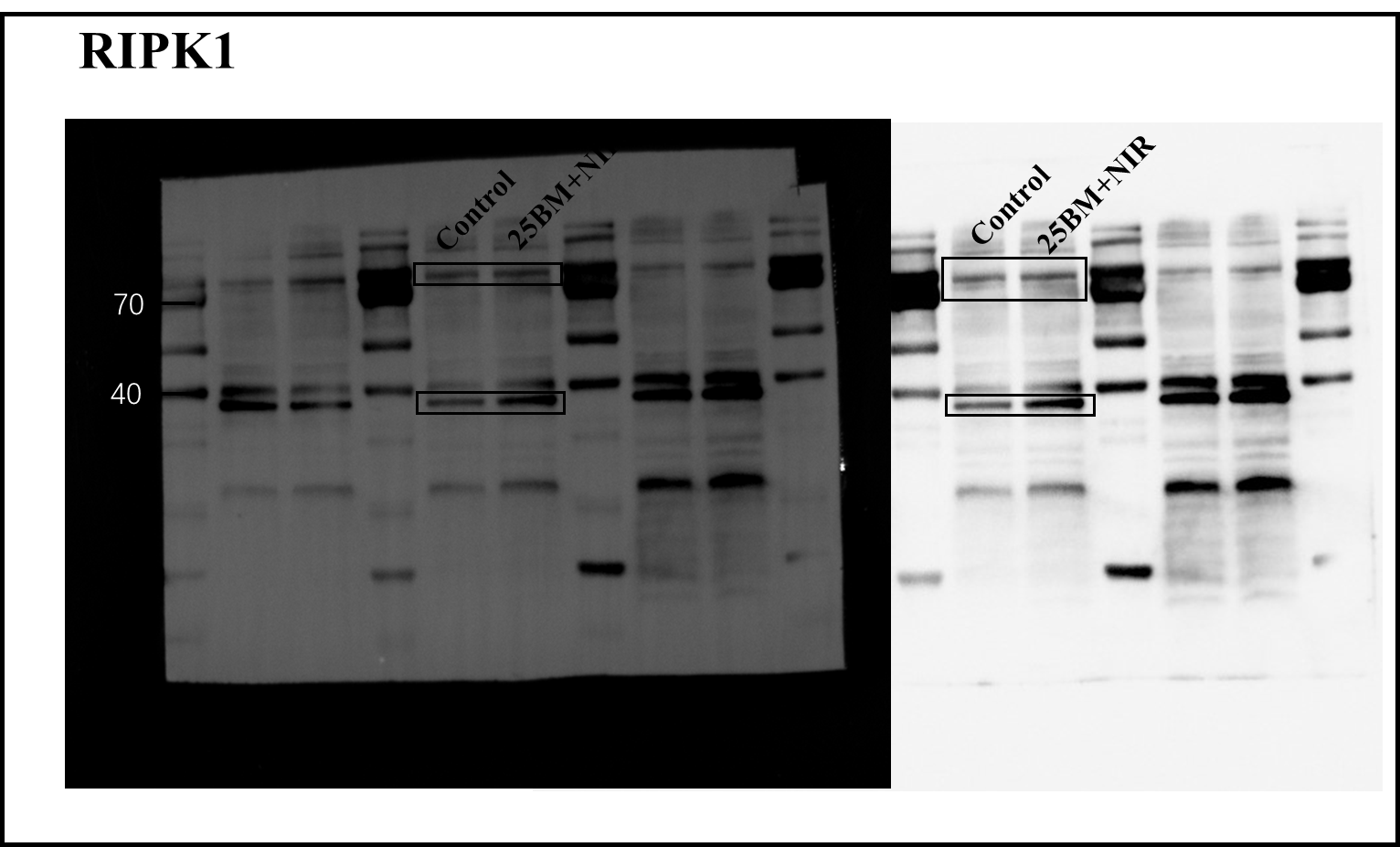


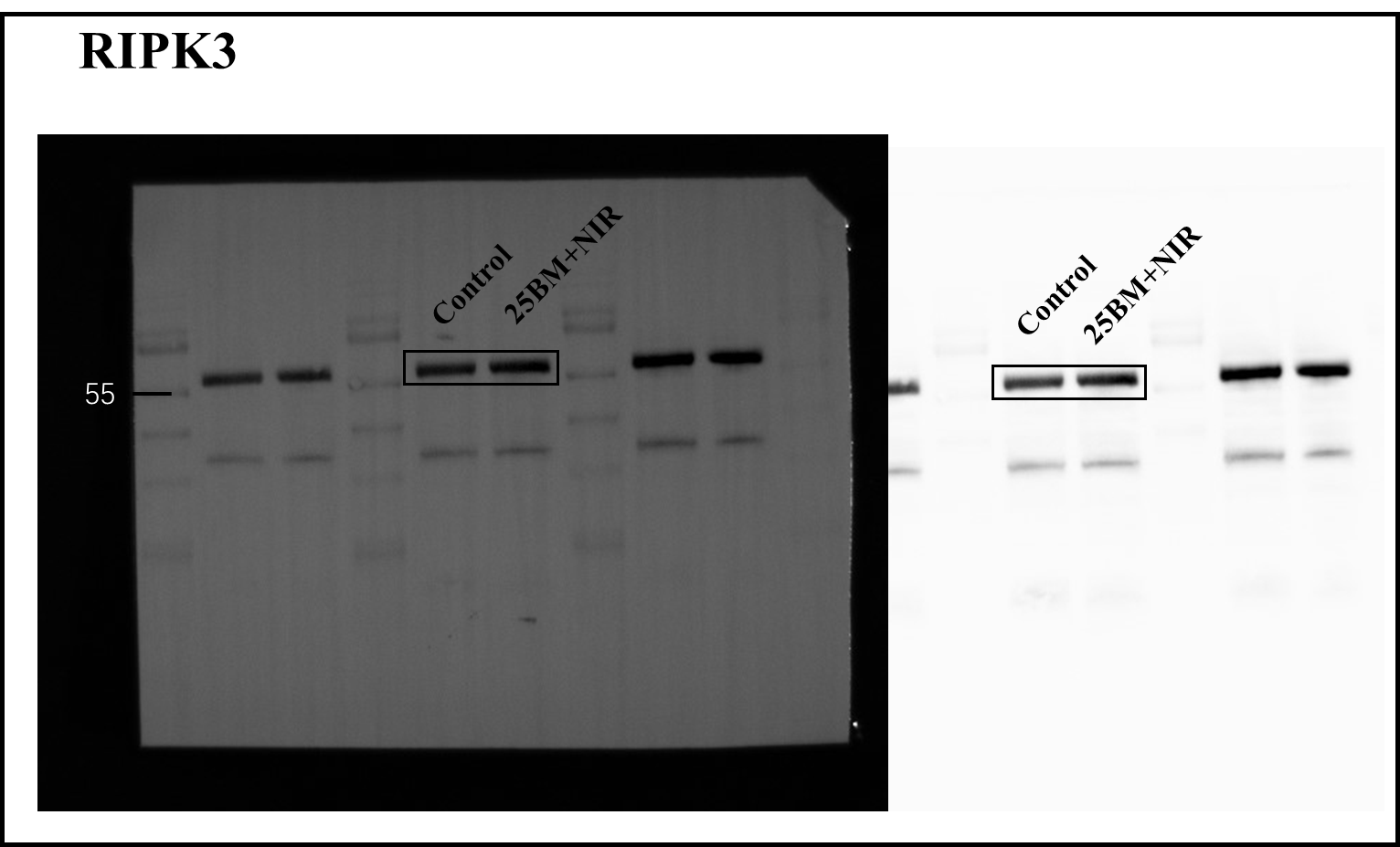


**Figure 5H**


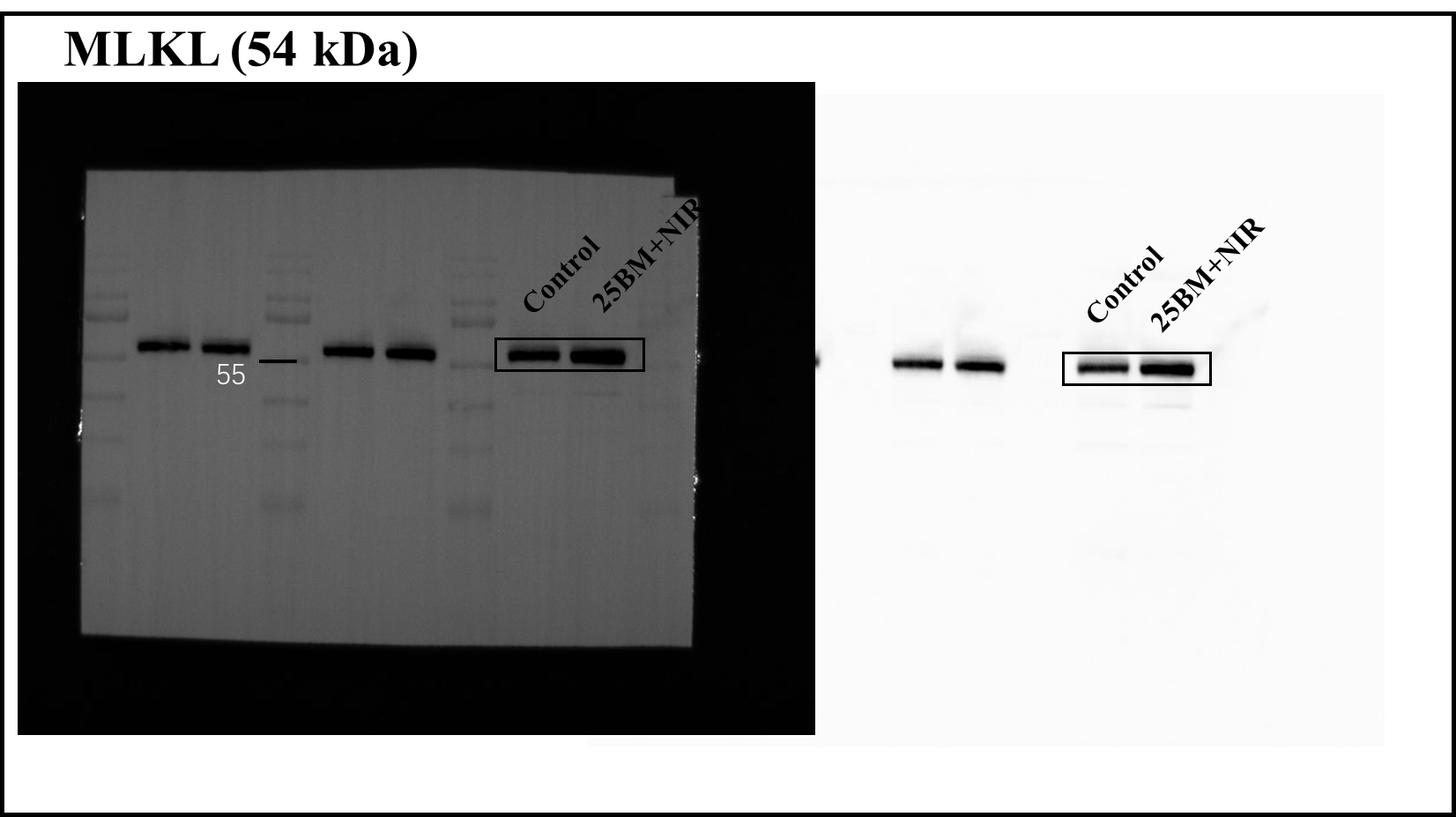


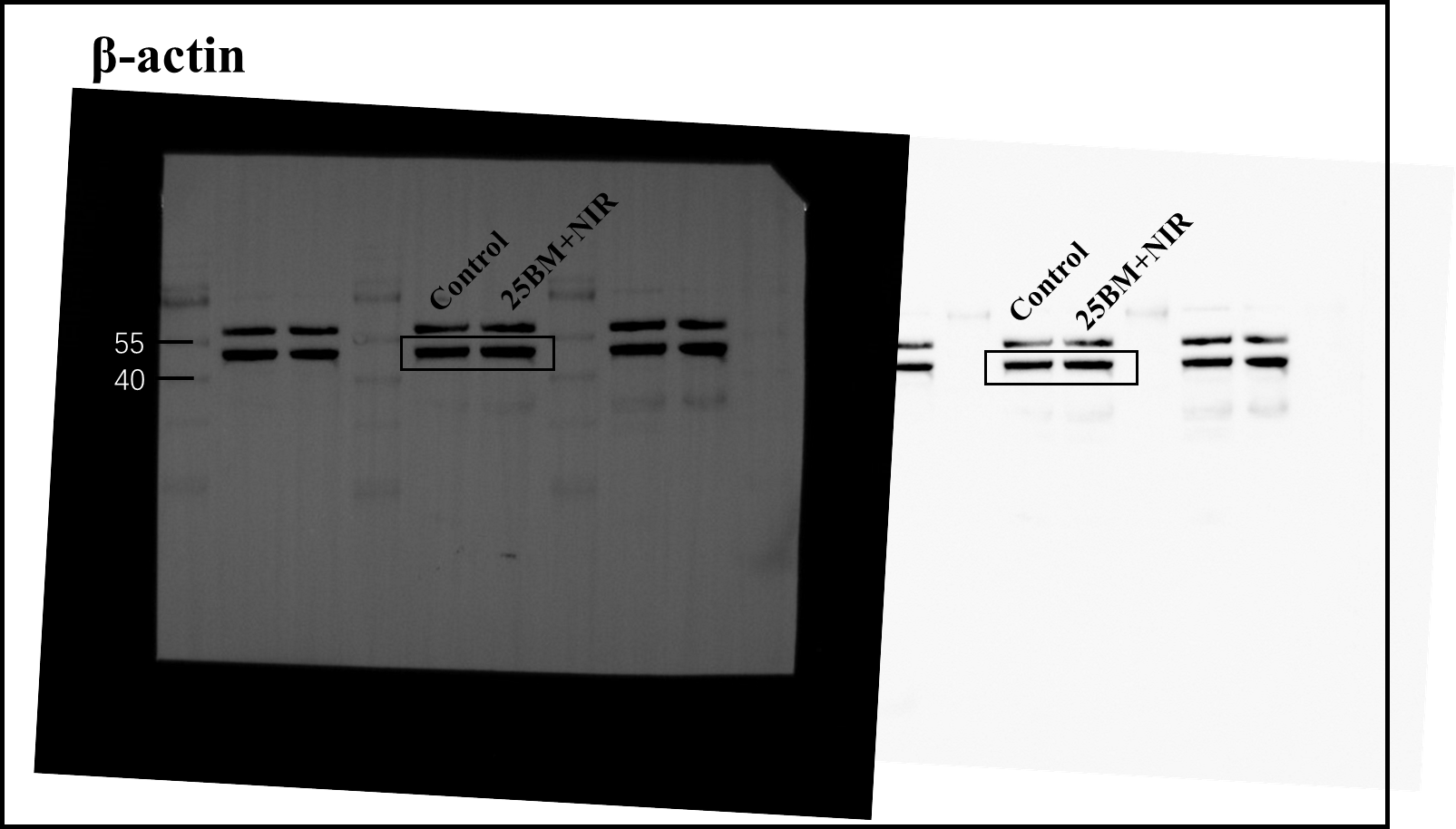

Supplement: Supplementary 1 — Figs. S1 to S11 Table S1 [file bmr.0228.f1.docx]
